# The AI-2/*luxS* Quorum Sensing System Affects the Growth Characteristics, Biofilm Formation, and Virulence of *Haemophilus parasuis*

**DOI:** 10.3389/fcimb.2019.00062

**Published:** 2019-03-19

**Authors:** Bingzhou Zhang, Xugang Ku, Xiaoqian Zhang, Yan Zhang, Guo Chen, Fangzhou Chen, Wei Zeng, Jing Li, Ling Zhu, Qigai He

**Affiliations:** ^1^State Key Laboratory of Agricultural Microbiology, Division of Animal Infectious Diseases, College of Animal Sciences and Veterinary Medicine, Huazhong Agricultural University, Wuhan, China; ^2^College of Animal Sciences and Veterinary Medicine, Xinjiang Agricultural University, Urumqi, China

**Keywords:** quorum sensing, *H. parasuis*, *luxS*, biofilm formation, virulence

## Abstract

*Haemophilus parasuis* (*H. parasuis*) is a kind of opportunistic pathogen of the upper respiratory tract of piglets. Under certain circumstances, virulent strains can breach the mucosal barrier and enter the bloodstream, causing severe Glässer's disease. Many virulence factors are found to be related to the pathogenicity of *H. parasuis* strain, but the pathogenic mechanism remains unclear. LuxS/AI-2, as a kind of very important quorum sensing system, affects the growth characteristics, biofilm formation, antibiotic production, virulence, and metabolism of different strains. In order to investigate the effect of luxS/AI-2 quorum sensing system on the virulence of *H. parasuis*, a deletion mutant strain (ΔluxS) and complemented strain (C-luxS) were constructed and characterized. The results showed that the *luxS* gene participated in regulating and controlling stress resistance, biofilm formation and virulence. Compared with wild-type strain, ΔluxS strain decreased the production of AI-2 molecules and the tolerance toward oxidative stress and heat shock, and it reduced the abilities of autoagglutination, hemagglutination, and adherence, whereas it increased the abilities to form biofilm *in vitro. In vivo* experiments showed that ΔluxS strain attenuated its virulence about 10-folds and significantly decreased its tissue burden of bacteria in mice, compared with the wild-type strain. Taken together, the luxS/AI-2 quorum sensing system in *H. parasuis* not only plays an important role in growth and biofilm formation, but also affects the pathogenicity of *H. parasuis*.

## Introduction

Quorum sensing system (QS) was firstly discovered and described in two luminous marine bacterial species, *Vibrio fischeri* and *Vibrio harveyi* and it was reported to have regulated gene expression in response to increasing cell population density through autoinducer molecules (Nealson and Hastings, 1979). Based on the difference in autoinducers, quorum sensing system is classified into four types. The first type is luxR-I quorum-sensing system, in which LuxI is responsible for the production of the N-acyl-homoserine-lactone (AHL) autoinducer, and LuxR is activated by this autoinducer to increase transcription of the luciferase operon (Waters and Bassler, 2004). The second type is autoinducer peptide (AIP, a kind of short peptide signaling) quorum sensing system that exists in gram-positive bacteria (Waters and Bassler, 2004). The third type is *luxS*/AI-2 quorum sensing system which exists in approximately half of all the sequenced bacterial genomes (Waters and Bassler, 2004), and also in both gram-negative and positive bacteria. The last type is AI-3/epinephrine/norepinephrine quorum sensing system (Kendall and Sperandio, 2007). As a very important regulating system, quorum sensing system is associated with a diverse array of physiological activities and abilities, such as symbiosis, virulence, conjugation, antibiotic production, motility, sporulation, and biofilm formation (Miller and Bassler, 2001).

*LuxS*, as a kind of enzyme, plays an important role in activated methyl cycle (AMC) which is a pivotal metabolic pathway that serves to recycle homocysteine from S-adenosyl methionine (SAM) to maintain the *de novo* methionine biosynthesis. The secondary product (DPD) of this reaction undergoes spontaneous cyclization to form a mixture of different furanones including AI-2 accumulated in the culture supernatant (Hardie and Heurlier, 2008). The *luxS*/AI-2 quorum sensing system is reported to exist in different strains, such as *Gamma* and *Betaproteobacteria, Lactobacillales*, and *Bacillales*, especially in many *Pasteurella* strains (Sun et al., 2004; Rao et al., 2016). But, the function of quorum sensing system varies in different strains. For example, the deletion of *luxS* gene tends to significantly decrease bacterial biofilm formation, cell adhesion, hemolytic activity, and transcription levels of some virulence genes in *Streptococcus suis* strain (Wang et al., 2011). In *H. influenzae, luxS* gene can inhibit biofilm formation and increase virulence (Armbruster et al., 2009; Pang et al., 2018). However, *luxS* gene exerts a completely opposite function in *A. pleuropneumoniae*, compared with its function in *H. influenzae* strain (Li et al., 2008). Therefore, the functions of *luxS* gene in different strains are significantly different.

*H. parasuis*, a member of the *Pasteurellaceae* family, can cause Glässer's disease that is characterized by severe infection of the upper respiratory tract, polyserositis, meningitis, and arthritis in pigs (Liu et al., 2016). Although *H. parasuis* can lead to a severe disease and huge economic losses, its pathogenic mechanism has not been very clear, yet. *H. parasuis* can be classified into at least 15 serotypes. Among these serotype strains, serotype 1, 5, 10, 12, 13, 14 strains are highly virulent, and serotype 2, 4, 15 strains are moderately virulent, and the rest are non-virulent (Jin et al., 2006).

It is very important to study the function of quorum sensing system and to reaveal its effect on growth characteristics and virulence of *H. parasuis*. However, so far, few reports about quorum sensing system in *H. parasuis* have been available. Therefore, this study is aimed to illustrate the function of quorum sensing system related gene *luxS* in *H. parasuis* by comparing, and evaluating the biological and virulent characteristics of wild-type HPS strain, *luxS* gene deletion mutant strain, and complemented strain.

## Materials and Methods

### Bacterial Strains, Plasmids, Primers, and Culture Conditions

The bacterial strains, plasmids, and primers used in this study are listed in Table 1. Standard reference strain of *H. parasuis* serotype 2 (HPS2) was grown in Tryptic Soy Broth (TSB) or Tryptic Soy Agar (TSA) medium (Difco Laboratories, Detroit, MI, USA) supplemented with 10 μg/mL of nicotinamide adenine dinucleotide (NAD) and 5% (v/v) inactivated cattle serum (T/V/S) (Zhejiang Tianhang Biotechnology, Zhejiang, China) at 37°C. The culture condition of mutant strain (ΔluxS) and complemented strain (C-luxS) are the same with that of wild-type strain with extra kanamycin (50 μg/mL) or gentamicin (20 μg/mL) (Sigma-Aldrich, Missouri, USA). *Escherichia coli* DH5α was grown in Luria–Bertani medium at 37°C. The *luxS* genes of 15 standard reference strains of *H. parasuis* strains were sequenced in GeneScript (Nan Jing, China).

**Table 1:** Characteristics of bacterial strains, plasmids, and primers used in this study.

**Strain, plasmid or primer**	**Characteristics and/or sequences**	**Source/References**
**STRAIN**		
*E. coli* DH5α	supE44 ΔlacU169 (ϕ80lacZΔM15) hsdR17 recA1 endA1 gyrA96 thi-1 relA1ΔluxS	Purchased from TaKaRa (Otsu, Japan)
*V. harveyi* BB170	AI-2 reporter strain (AI-1 sensor^−^, AI-2 sensor^+^)	Bassler et al., 1997
*V. harveyi* BB886	AI-1 reporter strain (AI-1 sensor^+^, AI-2 sensor^−^)	Bassler et al., 1997
*H. parasuis* 2 (HPS2)	Reference strain of serotype 2	Preserved in our lab Kielstein and Rapp-Gabrielson, 1992
*H. parasuis* 2ΔluxS::kan (ΔluxS)	luxS mutant of *H. parasuis* serotype 2, Kan^r^	This study
*H. parasuis* 2ΔluxS -luxS (C-luxS)	The complement of *H. parasuis* 2 ΔluxS::kan containing pSHK_3_- C- luxS, Kan^r^, Gm^r^	This study
**PLASMIDS**		
pK18mobsacB	Suicide and narrow-broad-host vector, Kan^r^	Zhang et al., 2012
pK18-ΔluxS::kan	A 2064 bp overlap fragment containing Kan^r^, the upstream and downstream sequences of the *luxS* gene in pK18mobsacB, Kan^r^	This study
pSHK_3_	*E. coli*–*H. parasuis* shuttle vector, Kan^r^	Wang et al., 2013
pSHK_3_-Gm	Kan^r^ replaced with Gm^r^ (534 bp) in pSHK3, Gm^r^	This study
pSHK_3_-C-luxS	A fragment containing the 660bp promoter and complete *luxS* ORF in pSHK3-Gm, Gm^r^	This study
**PRIMERS**		
HPS-LuxS-F/R	CCGGAATTCATGCCTTTACTAGATAGC; CGCGGATCCCTATGGATTTAGC; the amplified sequence of *luxS* gene	This study
H-LuxS-u F/R	CCGGAATTCACCGCTTGTTAATACCGAGTCCACCATTG; TTATCTTGTGCAATGAACGATTCTCCAATAAATA; the amplified flank sequence upstream *luxS* coding sequence (566 bp)	This study
Kan-F/R	TATTTATTGGAGAATCGTTCATTGCACAAGATAA; TTAGTTCCGTAGCAATAACAATTAACCAATTCTGATTAG; the amplified sequence of kanamycin resistance gene (909 bp)	This study
H-LuxS-d F/R	CTAATCAGAATTGGTTAATTGTTATTGCTACGGAACTAA; TGCTCTAGAACAAGCGGTAGCAGATACACGCCCAGC; the amplified flank sequence downstream *luxS* coding sequence (553 bp)	This study
HPS-16S-F/R	GTGATGAGGAAGGGTGGTGT; GGCTTCGTCACCCTCTGT; the amplified sequence of 16S rRNA gene of H. parasuis	Oliveira et al., 2001
C-LuxS-F/R	CCGGAATTCATACAGAATTTTGATTGAAC; TGCTCTAGACTATGGATTTAGCAATTTCTC; the amplified sequence of *luxS* gene and its promoter sequence	This study
hscA (0059)-F/R	CGCCATTAACCTCTATTGACCC; CTTCCATATCTTGCTTCGCATT; the amplified flank sequence upstream *luxS* coding gene “HAPS_0059”	This study
typA (0064)-F/R	ATGAATTAGCGGTATCTCGTCC; TACTTCGTGCTGGTACTTGTCG; the amplified flank sequence downstream *luxS* coding gene “HAPS_0064”	This study

### Homology Analysis of *luxS* Gene in Different Strains

The *luxS* gene of *H. parasuis* serotype 2 was compared with some representative strains currently available from the National Center for Biotechnology Information (NCBI) through Basic Local Alignment Search Tool (BLAST) program with the default settings (Sun et al., 2004). The detailed *luxS* gene sequences of the reference strains were listed in Supplementary Data. Multiple sequence alignments were performed by the maximum likelihood algorithm method (bootstrap analysis with 1,000 replicates) with MEGA Version 6.06 (Chen et al., 2017).

### Construction and Verification of *luxS* Deletion Mutant and Complemented Strains

All plasmids and primers used for the construction of *luxS* deletion mutant and complemented strains were listed in Table 1. The upstream (566 bp) and downstream (553 bp) fragments of *luxS* gene from HPS2 genome, and kanamycin resistance cassette (909 bp) from pSHK3 plasmid were amplified using primer pairs HPS-LuxS-u F/R, H-LuxS-d F/R, and Kan-F/R, respectively. The overlap extension method was used in these three fragments to construct a new fragment UKD (*luxS* upstream sequence, kanamycin resistance cassette sequence and *luxS* downstream sequence), then, the obtained UKD fragment was inserted into pk18mobsacB plasmid with EcoRI and XbaI restriction enzymes to generate recombinant plasmid pk18-UKD. The recombinant plasmid was introduced into HPS2 by natural transformation method as described in previous studies with a simple modification (Zhang et al., 2012; Wang et al., 2013; Zou et al., 2013). Briefly, 20 μL of cAMP (8 mM) was added to 20 μL of recipient bacterial suspension in logarithmic phase (OD_600_ = 0.9). Ten minutes later, 2 μg of donor DNA plasmid was added to the bacterial mixture. Afterwards, the cells were added to T/V/S plate and incubated at 37°C for 6 h. Subsequently, cells were transferred to a kanamycin selective plate. At last, the cells were incubated at 37°C for 48 h.

The complemented plasmid pSHK3-C-luxS was constructed by amplifying a 660 bp *luxS* open reading frame (ORF) and its promoter with primers C-LuxS-F/R. Subsequently, the amplicon was inserted into pSHK3-Gm plasmid derived from the framework of pSHK3-Kan with its kanamycin gene (909 bp) replaced by gentamicin gene (534 bp). The complemented plasmid was then introduced into the *luxS* deletion mutant by electroporation (2.5 kv, 5 ms) (Wang et al., 2013).

To verify the construction results of the deletion mutant and complemented strains, *luxS*, gentamicin gene, kanamycin gene, upstream (HAPS_0059) and downstream (HAPS_0064) genes of *luxS* were amplified and verified by sequencing.

### AI-1 and AI-2 Bioluminescence Assays

The assay was carried out based on the standard method described in the previous studies (Bassler et al., 1997; Wang et al., 2017) with some modifications. The bacteria for testing autoinducer product were grown in an orbital shaker at 30°C, then supernatant of different time point (from 0 to 16 h) was collected and cells were removed from the culture fluid by centrifugation at 5,000 × g for 5 min, followed by the passage of the culture fluids through 0.22 μm pore size membrane filters. Meanwhile, the OD_600_ values at different time points were also measured. *V. harveyi* BB170 (AI-1 sensor-, AI-2 sensor+) and BB886 (AI-1 sensor+, AI-2 sensor-) as reporter strains to detect AI-1 and AI-2 molecules were grown overnight at 30°C in AB medium. The cultured suspension was diluted 5,000 times in fresh AB medium. Subsequently, 90 μL of the diluted cells were added to microtitre wells, and 10 μL of cell-free culture fluid from the tested strains was added to each corresponding wells with a final concentration of 10%. The supernatant of the overnight culture of *V. harveyi* BB170 or BB886 was used as the positive control, and AB medium was used as the negative control. The plates were incubated at 30°C in the incubator. Luminescence was measured every hour using a Synergy TM HT Multi-Detection Reader (Bio Tek Instruments, USA). AI-2 activity was quantified as relative luminescence units (RLU) at the time when the negative control produced the smallest amount of luminescence.

### Growth Characteristics of Different Strains of *H. parasuis*

The growth characteristics of wild-type strain HPS2, mutant strain ΔluxS, and complemented strain C-luxS were measured (Huang et al., 2016). Three kinds of strains were grown in 6 mL T/V/S medium overnight and then diluted in the same medium to an OD_600_ value of 0.8. The 200 μL of the diluted suspension was inoculated into 200 mL of fresh T/V/S medium, and then incubated at both 37°C and 40°C, respectively in a shaker with 180 rpm. The OD_600_ value was determined using an Eppendorf Biospectrometer (Eppendorf, Hamburg, Germany) at 2 h intervals. The colony forming units (CFUs) were determined at 4 h intervals by counting colonies at the appropriate dilution obtained after a series of dilution. The experiments were performed in triplicate for three times.

### Stress Resistance Assays

Stress resistance assays were performed based on a previously described method (Xie et al., 2013; Huang et al., 2016) with some modifications. The OD_600_ value of overnight cultivated *H. parasuis* wild-type strain HPS2, mutant strain ΔluxS, and complemented strain C-luxS was adjusted to 0.8. In the oxidative stress tolerance assay, 500 μL of 1 M hydrogen peroxide was added to 500 μL of cell suspension and incubated at 37°C for 30 min. In the heat-shock assay, cells were incubated in a 48°C water bath for 30 min. Untreated cell suspensions of each strain after the incubation at 37°C for 30 min were used as a control in each experiment. Following incubation, the cultures were serially diluted by PBS, and their CFUs were determined by plate counting. The proportion of stress-resistant cells to control cells was calculated as (CFU/mL in stress group / CFU/mL in control group) × 100%. Each assay was independently performed in triplicate for three times.

### Biofilm Formation Assay

Biofilm formation assay was conducted on 96 wells microtiter plates (Thermofisher, USA) based on the methods described in previous studies (Stepanovic et al., 2000; Jin et al., 2006) with some modifications. The OD_600_ value of overnight cultivated strains was adjusted to 0.8 with the same treatment in the growth analysis assay. The 20 μL of inoculum was added to each well which contained 180 μL T/V/S medium, then was incubated at 37°C for different hours (12, 24, 36, 48, and 60 h). Each strain was tested in triplicate. After the time, the liquid of each well was removed with an injector and then the wells were washed three times with 200 μL sterile PBS to remove loosely adherent cells. The remaining bacteria attached to wells were fixed with 100 μL of methanol for 30 min. After being dried in air, the wells were stained with 200 μL of 1% crystal violet solution for 10 min at room temperature. Excess crystal violet was removed from the wells. Afterwards, the wells were washed to make sure the flowing water clean. Thereafter, the plates were dried in a 37°C incubator for 30 min and the dry cells were dissolved with 100 μL of 33% (v/v) glacial acetic acid, and the OD_630_ value of each well was measured with the same instrument as used in the detection of luminescence. All tests were carried out in sextuplicate for three times, and results were averaged. The wells that were not inoculated with bacteria were used as negative controls.

### Autoagglutination Assay

Autoagglutination ability of *H. parasuis* strains was determined using a previously described method (Labandeirarey et al., 2010; Zou et al., 2013). Briefly, bacteria overnight cultivated to stationary phase were transferred into sterile tubes, OD_600_ of three kinds of strains was adjusted to the same value, and the tubes remained static at different environment temperatures (4, 25, 37°C). At last, the OD_600_ value was measured every hour in subsequent 24 h. All tests were performed in triplicate for three times and the results were averaged.

### Hemagglutination Assays

Overnight cultivated strains with their OD_600_ value adjusted to 0.8 were centrifuged at 6,000 rpm for 5 min and re-suspended in PBS. A 50 μL of suspension and its 2-fold serially diluted counterpart were added to a 96-well V-bottom Costar polypropylene plate (Fisher Scientific Co., USA) in sextuplicate. The 2% (vol/vol) suspension of erythrocytes derived from healthy swine was prepared using PBS. A 50 μL of the erythrocyte suspension was then added to each well, and the last line of the wells was used as negative control only with erythrocyte suspension. The microtiter plate was gently agitated on a vortex mixer for 30 s. Hemagglutination was recorded photographically after the incubation of the plate at 37°C for 30 min (Pearson et al., 2002). All of the above assays were performed in sextuplicate for three times.

### Adhesion Assays

Adhesion assays were performed using porcine kidney epithelial cells (PK-15) (Zhang et al., 2012, 2014) following a previously described method (Vanier et al., 2004, 2006). Briefly, the cells (5 × 10^5^) were seeded onto 24-well tissue culture plates in Dulbecco's Modified Eagle's Medium (DMEM, Invitrogen) containing 10% heat-inactivated fetal bovine serum (Suero, Industria Argentina). After cells were cultured at 37°C in a humidified incubator at 5% CO_2_ for 24 h, the tissue culture cells were washed thrice with PBS and infected with approximately 10^7^ CFU *H. parasuis*. Culture plates were incubated at 37°C for up to 2 h to allow bacterial adhesion. Cells were rigorously washed five times with PBS to eliminate non-specific bacterial attachment and then incubated with 100 μL 0.25% trypsin/EDTA at 37°C for 10 min. After the incubation, the cells were re-suspended from the bottom of every well. The cell suspensions with adherent bacteria were diluted 10-folds and put onto TSA plates containing NAD and serum. The adhesion rate was calculated as the bacteria adhered to cells dividing the added bacteria × 100%. All of the above assays were performed in triplicate for three times.

### Determination of the 50% Lethal Dose (LD_50_)

The virulence of wild-type strain HPS2, mutant strain ΔluxS, and complemented strain C-luxS was evaluated using 18–20 g female Balb/C mice (Li et al., 2017; Zhao et al., 2017) which were purchased from Huazhong Agricultural University animal center. A total of 130 mice were randomly divided into 13 groups with 10 mice in each group. The wild-type strain HPS2, mutant strain ΔluxS, and complemented strain C-luxS were cultured at 37°C until the last stage of logarithmic phase. Cells were collected from the culture fluid by centrifugation at 5,000 × g for 8 min, washed 3 times with PBS, and re-suspended in PBS. Then, the 3 types of strains were diluted with each strain corresponding to 4 concentrations: 9.63 × 10^7^, 2.08 × 10^8^, 4.47 × 10^8^, and 9.63 × 10^8^ CFU/0.5 mL (wild-type strain HPS2), 7.57 × 10^8^, 1.55 × 10^9^, 3.34 × 10^9^, and 7.57 × 10^9^ CFU/0.5 mL (mutant strain ΔluxS), and 5.83 × 10^8^, 1.17 × 10^9^, 2.25 × 10^9^, and 5.83 × 10^9^ CFU/0.5 mL (complemented strain C-luxS), respectively. The mice were raised for 3 days before experiment for their adaptation to the environment. The experimental mice were injected intraperitoneally (i.p.) with 0.5 mL of the suspension. The control mice were injected with 0.5 mL PBS. The number of surviving mice was recorded for 14 days after infection and the LD_50_ value was calculated according to Karber's method (Li et al., 2017; Zhao et al., 2017). Anesthetic was used on the remaining mice before they were executed. The research was approved by the Ethics Committee of the Faculty of Veterinary Medicine of the Huazhong Agricultural University with the protocol number as 42816300002256. All procedures followed the instruction of the care and use of laboratory animals provided by Hubei provincial public service facilities.

### Determination of Viable Bacteria in Mice Organs

A total of 48 female Balb/C mice were randomly and averagely assigned to four groups and used for assessing the presence of viable bacteria in infected mice organs. The experimental mice were injected i.p. with 0.5 mL of HPS2, ΔluxS, or C-luxS (10^8^ CFU) strains, and the control mice were injected with 0.5 mL of PBS. The tissue samples (0.1 g/organ) of heart, liver, spleen, lung, and kidney were collected every day 1–4 days after infection and were fragmented into small pieces by using tissue homogenizer. After 10-fold serial dilution with PBS, the 100 μL of tissue mixtures at different dilution concentration were plated onto TSA plates and incubated at 37°C for 48 h. The number of colonies were counted and presented as CFU/1 g. When the number of bacteria in every kind of tissue was counted, three plates were used at every dilutability and each experiment was performed in triplicate (Li et al., 2017; Zhao et al., 2017).

### Statistical Analysis

The results are presented as the means ± standard deviation (SD). The statistical analysis was performed using the two-way ANOVA in Graph Pad Prism 7.0 (GraphPad Software Inc., USA). The significant difference was defined as ^*^p < 0.05, and the various degrees of significant difference were designated as ^**^p < 0.01, ^***^p < 0.001, ^****^p < 0.0001, respectively.

## Results

### The Homology of *luxS* Gene in Different Strains

**Figure 1:**
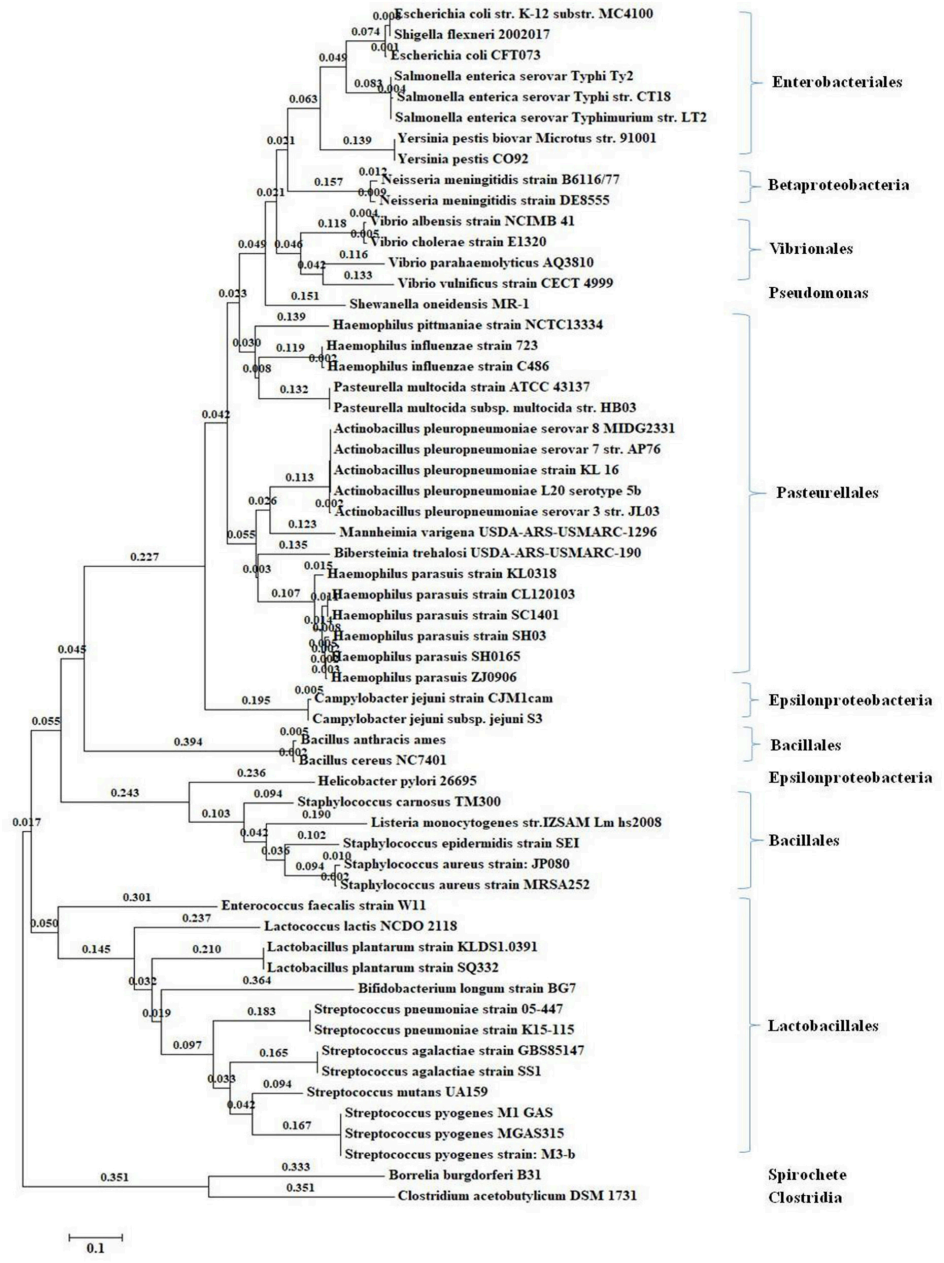
Phylogenetic tree of *luxS* gene from the completely sequenced genomes in the KEGG genome database. The maximum likelihood algorithm method with the GTR nucleotide substitution model was used when constructed the phylogenetic tree (bootstrap analysis with 1,000 replicates).

The sequences of *luxS* orthologs were aligned and a phylogenetic tree was built from the alignment. Phylogenetic analyses were performed to evaluate the genetic relationship between *H. parasuis* and heterologous species strains (Figure 1) or the same family strains (Supplementary Table 1). The phylogenetic analysis results showed that *luxS* gene served as a distinction mark among different species. There were mainly three bigger branches in the phylogenetic tree including *Gamma* and *Betaproteobacteria, Lactobacillales*, and *Bacillales* (Figure 1). *LuxS*, as an important quorum sensing gene, also commonly existed in *Pasteuriaceae* including *H. pittmaniae, H. influenzae, Pasteurella multocida, A. pleuropneumoniae, Mannheimia varigena, Bibersteinia trehalosi*, and *H. parasuis*. The nucleotide homology across several common *Pasteuriaceae* strains ranged from 69 to 78% and protein homology ranged from 73 to 83% (Supplementary Table 1), which indicated a great functional similarity. Furthermore, the homology of *luxS* gene among 15 standard reference strains of *H. parasuis* was over 95%, no matter at nucleotide or protein level (Supplementary Table 2).

### Construction and Verification of *luxS* Mutant Strain and the Complemented Strain

The PCR identification results of HPS2, ΔluxS, and C-luxS strains were shown in Supplementary Figure 1A 821 bp 16S rRNA fragment was identified in all three strains (Supplementary Figure 1A). The amplification of the fragments containing *luxS* gene, gentamicin gene, kanamycin resistance cassette sequence and UKD sequence produced the bands with different sizes (Supplementary Figures 1B–E), respectively. *LuxS* gene was found to exist in HPS2 and C-luxS strains. Gentamicin gene only existed in C-luxS strain. Kanamycin resistance cassette sequence existed in ΔluxS and C-luxS strains and UKD sequence. The different sizes of above-mentioned genes were observed in ΔluxS, C-luxS, and HPS2 strains. On the other hand, sequencing results indicated that plasmids, ΔluxS, and C-luxS strains exhibited no mutation. These results indicated the successful construction of *luxS* gene deletion mutant and its complemented strain.

In addition, the possible polarity effect resulting from wild-type HPS2, deletion mutant ΔluxS, and complemented strain C-luxS was examined by verifying the transcription of genes flanking *luxS* through RT-PCR (Supplementary Figure 1F). RT-PCR results showed that both upstream gene (HAPS_0059) and downstream gene (HAPS_0064) all exited in cDNA and DNA of HPS2, ΔluxS, and C-luxS genomes. Therefore, it could be inferred that the transcription was not affected by the deletion of *luxS* gene.

### Analysis of AI-1 and AI-2 Activity

**Figure 2:**
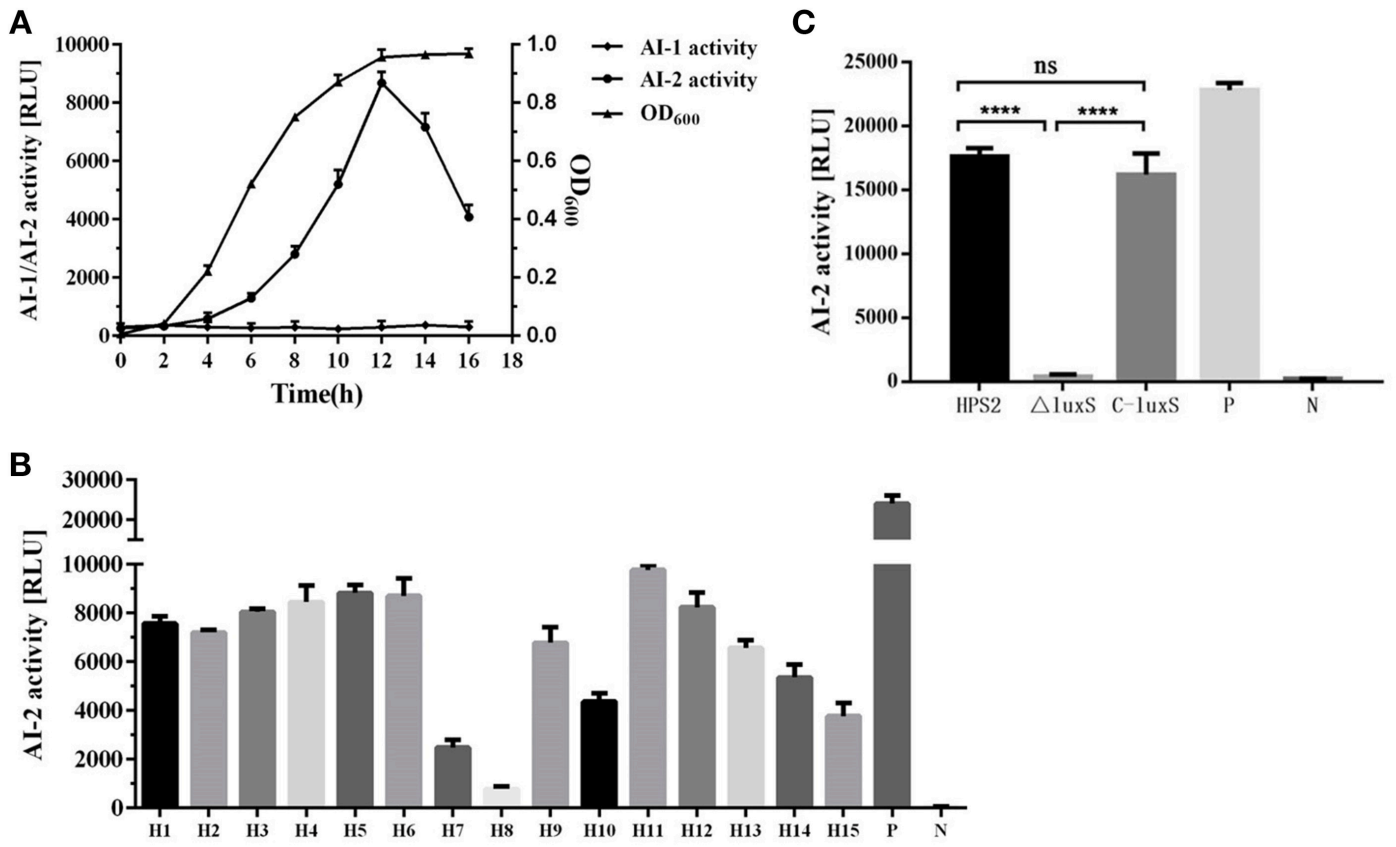
The activity of AI-1 and AI-2 molecules in wild-type strain HPS2, deletion mutant ΔluxS and complemented strain C-luxS was measured through BB170 and BB886 reporter strains. **(A)** The luminescence value of supernatant and OD_600_ value of HPS2 at different time points (0, 2, 4, 6, 8, 12, 14, 16 h). **(B)** The ability of *H. parasuis* serotype 1–15 to secrete AI-2 molecule. H1–H15 represent *H. parasuis* from serotype 1–15, respectively. P represents positive control, N represents negative control. **(C)** The luminescence value of AI-2 molecules in the supernatant of HPS2, ΔluxS, and C-luxS strains. P represents positive control, N represents negative control. In these three independent experiments, the supernatant of *V. harveyi* BB170 (V. h) was used as the positive control, and sterile AB medium (AB) was used as the negative control. The supernatant of HPS2, ΔluxS, and C-luxS strains were collected when the OD_600_ value reached 0.8. The incubation time of BB170 reporter strain was 5 h. All of the above assays were performed in triplicate for three times. Bars represent the mean ± standard deviation of three independent experiments. Statistical analyses were performed using the two-way ANOVA. ****p < 0.0001 represents the highest degree of significant difference, and ns means no statistic significance.

The ability of wild-type strain HPS2, deletion mutant ΔluxS, and complemented strain C-luxS to secrete AI-1 and AI-2 molecules was measured through BB170 and BB886 reporter strains. The result demonstrated that AI-2 molecule accumulation of was observed when BB170 was used as a reporter strain, while the similar accumulation was not observed in BB886 strain, suggesting that AI-1 molecule was not present in 15 kinds of standard serotype *H. parasuis* strains (no data available). Based on the luminescence values at different time points, it can be concluded that the strongest activity of AI-2 molecule in HPS2 was available after 12-h cultivation in the shaker (Figure 2A). To verify whether AI-2 molecule was a molecule commonly existed in different serotype *H. parasuis*, the supernatant of 15 kinds of standard reference strains of *H. parasuis* was detected using BB170 strain. A very strong activity of AI-2 molecule was observed in almost all serotype strains (Figure 2B) except in serotype 8 strain. As seen in Figure 2C, AI-2 molecule showed much stronger activity in HPS2 strain than in ΔluxS strains and negative control, and AI-2 molecule was not available in ΔluxS strain. However, the complemented strain successfully recovered the activity of AI-2 molecule (Figure 2C).

### Growth Characteristics of HPS2, ΔluxS, and C-luxS Strains Under Different Conditions

**Figure 3:**
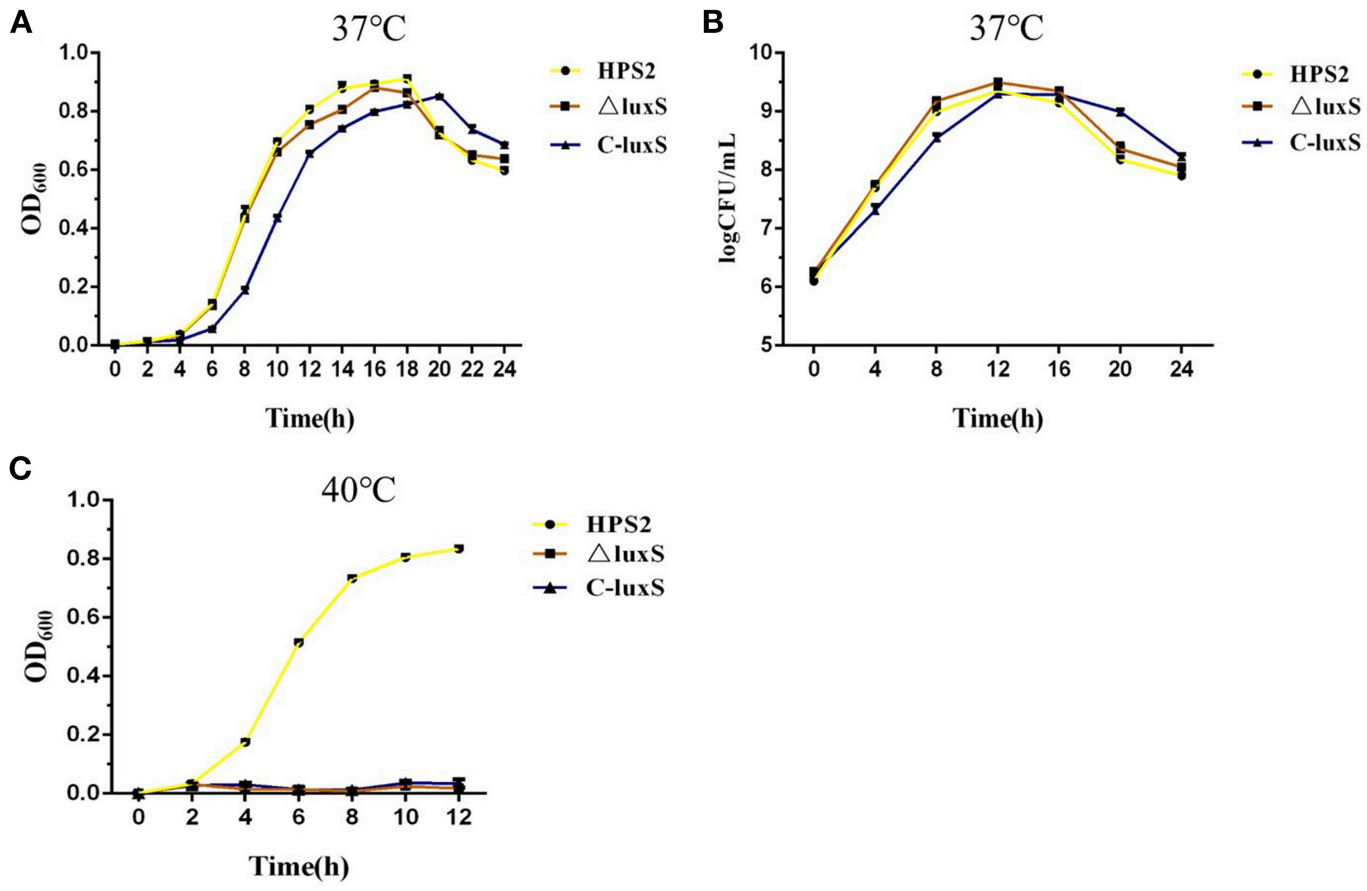
Growth analysis of the wild-type strain (HPS2), deletion mutant (ΔluxS), and complemented strain (C-luxS). Overnight cultures of HPS2 (•), ΔluxS (■), and C-luxS (▴) strains were diluted in T/V/S medium and then incubated at either 37°C **(A,B)** or 40°C **(C)**. Bacterial growth was monitored by measuring optical density at 600 nm **(A,C)** and viable cells at multiple time points **(B)**. The dots represent the mean value of three replicates, and error bars indicates standard deviations.

The growth characteristics of HPS2, ΔluxS, and C-luxS strains were investigated at 37 and 40°C, respectively. The results showed that the growth rate of HPS2 and ΔluxS strains was almost the same, while the growth of C-luxS strain was about 2 h later than that of wild-type and deletion mutant strains at 37°C, and the largest OD_600_ value of these three strains was almost the same. It took about 4 h for all three kinds of strains to enter logarithmic phase, while it took 12 h for HPS2 and ΔluxS strains to reach the stationary phases and 14 h for C-luxS strain. After about 18 h (HPS2 and ΔluxS strains) or 20 h (C-luxS strain) of incubation, the OD_600_ value of these 3 strains decreased significantly as time went on (Figure 3A). The cell counting results showed that three types of strains had almost the same number of viable cells, At 12 h, the highest CFU value was available for three strains: 2.25 × 10^9^CFU/mL for HPS2 strain, 3.15 × 10^9^CFU/mL for ΔluxS strain, and 2.01 × 10^9^CFU/mL for C-luxS strains, respectively (Figure 3B).This study found that these 3 strains displayed a significantly different growth characteristics at 40°C, that the growth of HPS2 was much faster than that of other 2 strains at 37°C, and that the growth of ΔluxS and C-luxS strains was inhibited (Figure 3C) at 40°C.

### Stress Resistance Results

**Figure 4:**
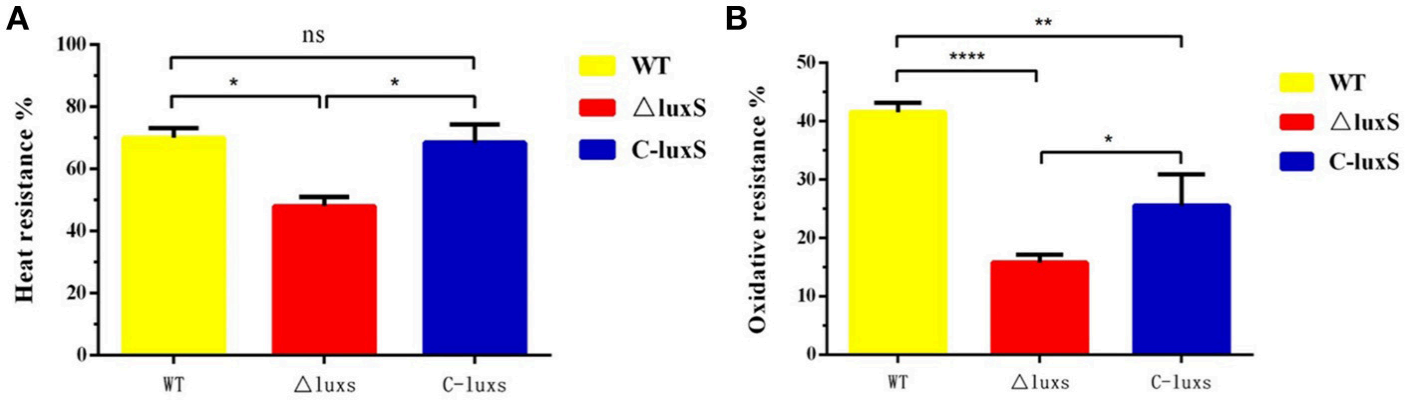
Analysis of the stress tolerance of the HPS2, ΔluxS, and C-luxS strains. Overnight cultured bacteria were diluted with OD_600_ value adjusted to 0.8. The 500 μL of each cell suspension was incubated in a 48°C water bath for 30 min **(A)** or treated with 500 μL of 1 M hydrogen peroxide **(B)** at 37°C for 30 min, respectively. All of the above assays were performed in triplicate for three times. Bars represent the mean ± standard deviation of three independent experiments. Statistical analyses were performed by using the two-way ANOVA. *p < 0.05, **p < 0.01, and ****p < 0.0001 represent increasing degrees of significant differences, respectively, and ns means no significant difference.

HPS2, ΔluxS, and C-luxS strains were exposed to various stress conditions, including heat shock and oxidative stress. When cells were treated in a 48°C water bath for 30 min, the survival rate of ΔluxS strain (48.1%) was obviously lower than HPS2 strain (70.1%) (*p* < 0.05). C-luxS strain (68.1%) showed a similar survival rate to HPS2 strain (Figure 4A). Similar pattern of survival rate was also found in oxidative stress assay. When cells were treated with 500 mM hydrogen peroxide for 30 min at 37°C, about 85% bacteria of ΔluxS strain were killed, but about 40% bacteria of HPS2 strain (*p* < 0.01) and 25% bacteria of C-luxS strain (*p* < 0.05) survived (Figure 4B). Taken together, these findings suggested an important role that *luxS* gene played in responding to various environment changes.

### Biofilm Formation in *H. parasuis enhanced by LuxS* Mutant Strain

**Figure 5:**
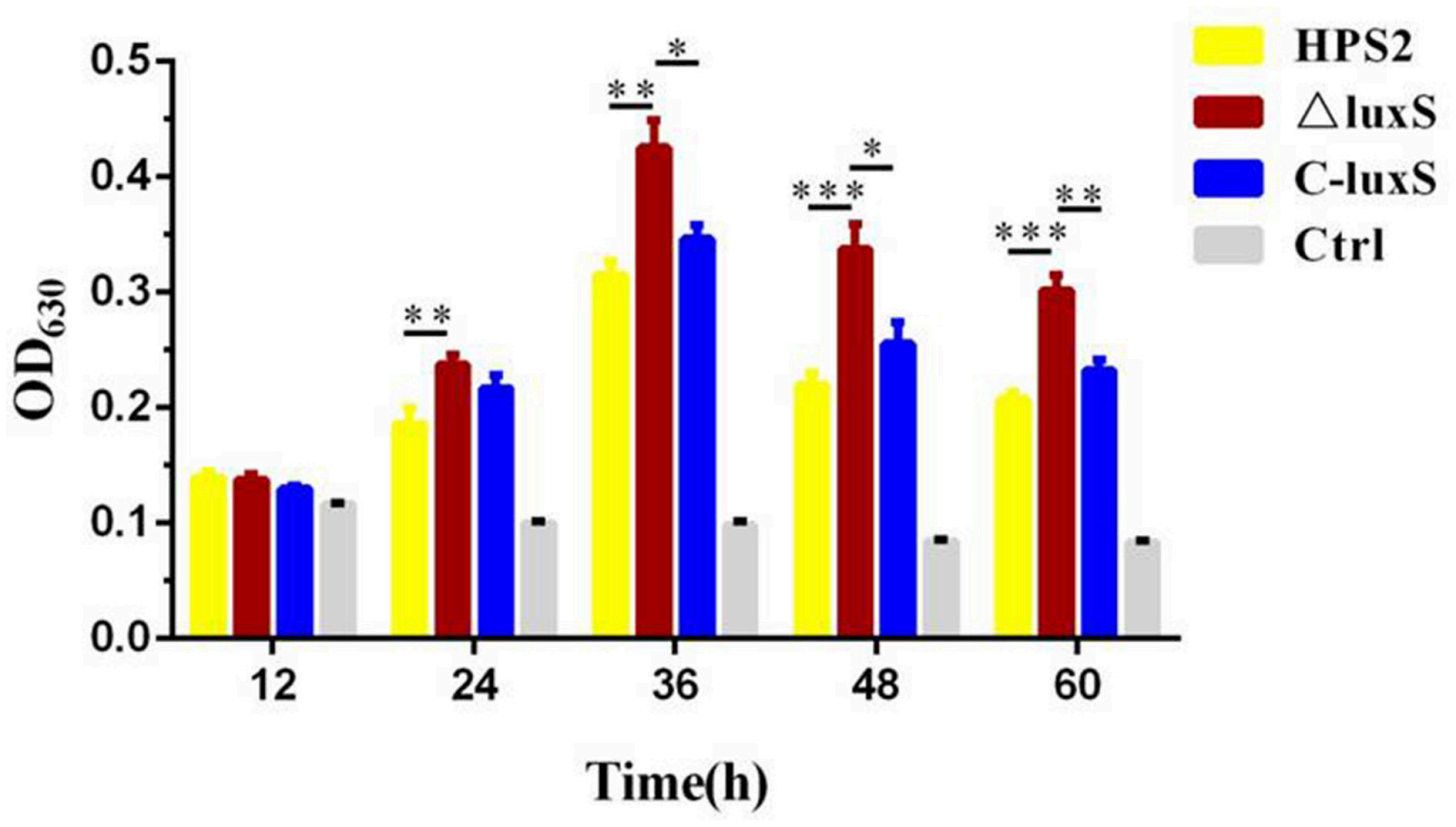
Biofilm formation ability of HPS2, ΔluxS, and C-luxS strains at different time points. Biofilm formation was determined by measuring the OD_630_ value of dissolved crystal violet. The biofilm formation ability was monitored by measuring OD_630_ value at 12, 24, 36, 48, and 60 h post incubation. Each strain was tested in 6 wells in a 96-well microtiter plate. Y-axis meant the relative biofilm value normalized as the OD_600_ value of the bacterial cultures. All above assays were performed in triplicate for three times. Bars represent the mean ± standard deviation of three independent experiments. Statistical analyses were performed using the two-way ANOVA. *p < 0.05, **p < 0.01, and ***p < 0.001 represent increasing degrees of significant difference.

To investigate the differences in biofilm formation among wild-type strain HPS2, deletion mutant ΔluxS, and complemented strain C-luxS under the same culture conditions, biofilm formation was quantitatively analyzed using microtiter plate assay at different time points. At the beginning of the first 12 h, no biofilm formation was observed at the bottom of microtiter plates. However, after 24 h of cultivation in T/V/S medium, an obvious biofilm formation was observed in HPS2, ΔluxS and C-luxS strains, and at 36 h, the largest amount of biofilm was observed. A significant difference in biofilm formation was found between HPS2 and ΔluxS strains, suggesting that ΔluxS strain could enhance biofilm formation ability from 24 to 60 h (*p* < 0.01), and that no significant difference in the biofilm formation ability was observed between C-luxS and HPS2 strain, so the complemented strain can restore biofilm formation ability to wild-type strain levels (Figure 5).

### Significantly Decreased Autoagglutination Ability of ΔluxS Mutant Strain

**Figure 6:**
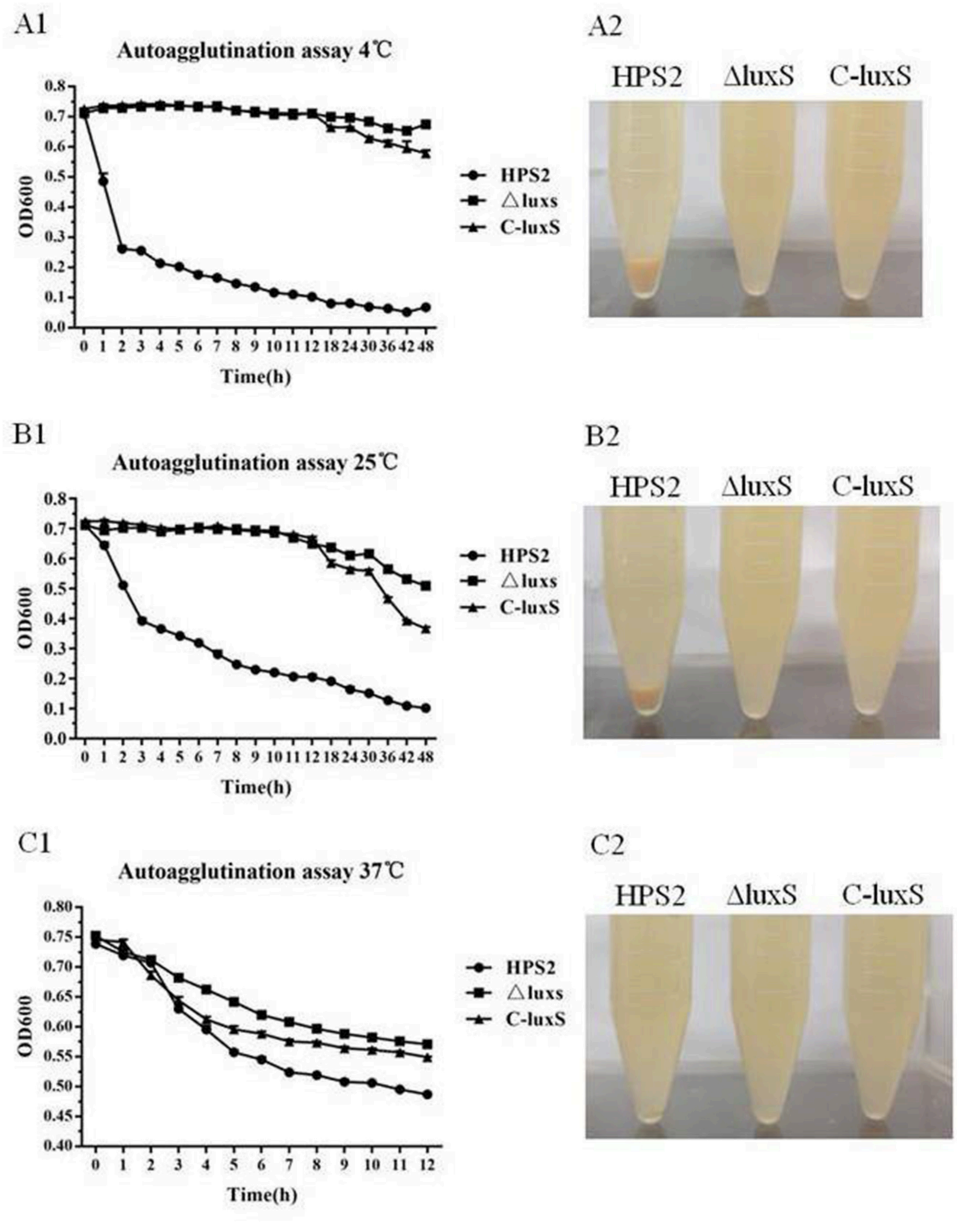
Autoagglutination ability of HPS2, ΔluxS, and C-luxS strains at different temperatures. Overnight cultured bacteria were diluted to an OD_600_ value near 0.75. The 0.2 mL of the upper liquid was taken to measure OD_600_ value from 1 to 24 h post-inoculation at 4°C **(A1,A2)**, 25°C **(B1,B2)**, and 37°C **(C1,C2)**. All of the above assays were performed in triplicate for three times. Bars represent the mean ± standard deviation of three independent experiments. Statistical analyses were performed using the two-way ANOVA.

The autoagglutination ability of HPS2, ΔluxS, and C-luxS strains were measured under different conditions. Based on the data, it can be concluded that the autoagglutination ability of ΔluxS and C-luxS strains was obviously weaker than that of HPS2 strain at 4, 25, or 37°C. The difference in autoagglutination ability between HPS2 and ΔluxS strains became increasingly obvious with temperature declining from 37 to 4°C. At 37°C, the difference in autoagglutination ability between HPS2 and ΔluxS strains was observed after about 3 h of culturation in an incubator. Later, autoagglutination ability was partially recovered in C-luxS strain (Figure 6A1). However, at 4°C and at 25°C, HPS2 and ΔluxS strains displayed a significant difference in autoagglutination ability after 1 h of culturation, and the partial recovery phenomenon was only found after 12 h of culturation (Figures 6B1, C1). In summary, HPS2 and ΔluxS strains displayed a significant difference in the autoagglutination ability at the first hour (4 and 25°C) (*p* < 0.001) or the third hour (37°C) (*p* < 0.001) post culturation. On the other hand, an autoagglutination phenomenon was directly observed, when the strains were cultured at 4, 25, and 37°C at the 12th h, respectively (Figures 6A2, B2, C2).

### Decreased Hemagglutination Ability of ΔluxS Strain

**Figure 7:**
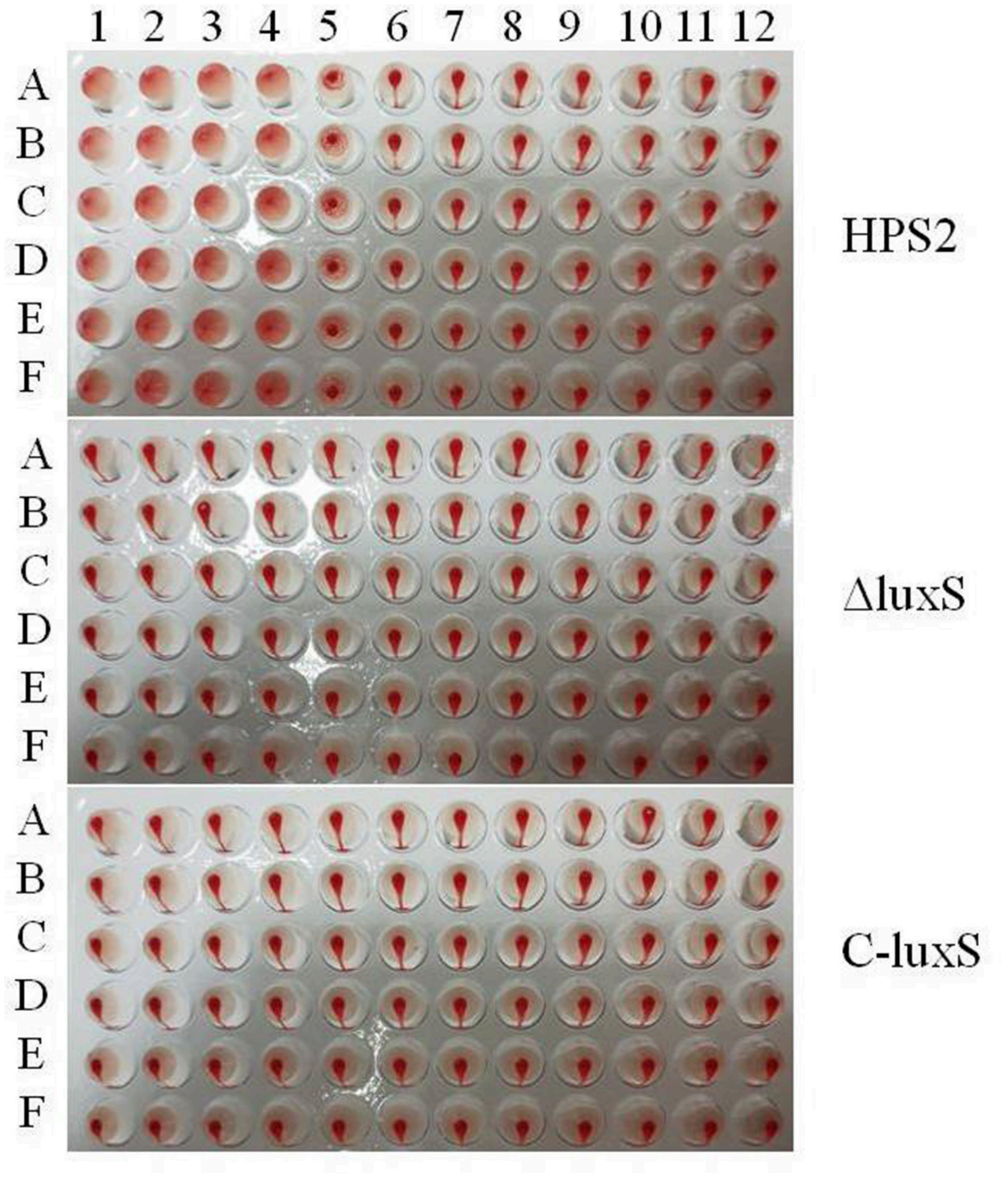
Hemagglutination ability of HPS2, ΔluxS, and C-luxS strains. Bacteria from line 1 to 11 were serially diluted. The last line (12) acted as the negative control (porcine red blood cells). The plates were incubated at 37°C for 30 min. All of the above assays were performed in sextuplicate (A–F) for three times.

To examine the hemagglutination ability of HPS2, ΔluxS, and C-luxS strains, porcine red blood cells were used to detect hemagglutination titer. The hemagglutination titer of HPS2 strain was found to be 2^5^, in contrast, no hemagglutination ability of ΔluxS and C-luxS strains was observed, even if 10^8^ CFU bacteria were added (Figure 7). Therefore, it could be inferred that the decrease in hemagglutination ability of HPS2 strain might be attributed to the deletion of *luxS* gene.

### Effect of *luxS* Gene on *H. parasuis* Adherence Ability in PK-15 Cells

**Figure 8:**
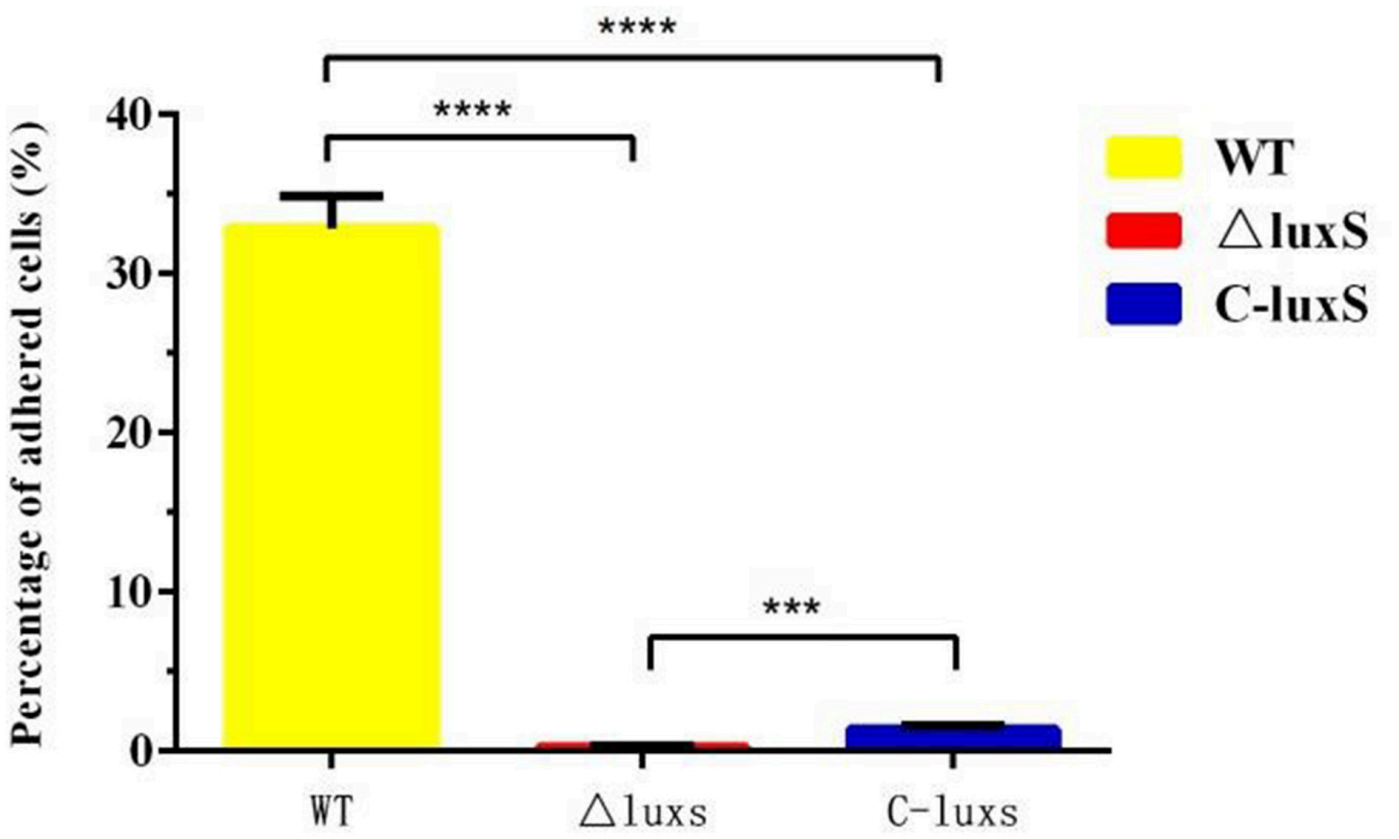
Adherence ability of HPS2, ΔluxS, and C-luxS strains in pk-15 cells. The percentage of adhered cells represents bacteria adhered to cells in each well of a 24-well plate. Culture plates were incubated for up to 2 h at 37°C. Error bars represent the standard deviation of three independent experiments performed in triplicate. Statistical analyses were performed using the two-way ANOVA. ***p < 0.001 and ****p < 0.0001 represent higher and the highest degree of significant difference, respectively.

To determine whether *luxS* gene interacted with host cells, porcine kidney cells (PK-15 cells) were utilized to compare the adherence ability of HPS2, ΔluxS, and C-luxS strains. After incubation at 37°C for 2 h, about 33% HPS2 strains adhered to pk-15 cells, while only about 0.28% ΔluxS strains adhered to pk-15 cells. Thus, the adherence ability of HPS2 strain was more than 100 times higher than that of ΔluxS strain. Meanwhile, the adherence ability of C-luxS strain was about 1.4%, which was 5 times as high as that of ΔluxS strain. Therefore, adherence ability was partially recovered in the complemented strain (Figure 8).

### *LuxS* Gene Was Associated With the Virulence of *H. parasuis*

The effect of the *luxS* gene on virulence was evaluated using Balb/C mouse model i.p. injected with various doses of HPS2, ΔluxS, and C-luxS strains. The mortality of mice was observed within 14 days after challenging. Depression, rough coat, tremble, and prostration were observed in high dose group mice after 6 h of injection, and most of mice died within first 3 days. The final LD_50_ value of HPS2 was found to be 4.46 × 10^8^ CFU, which the value for the same effect in ΔluxS strain was 4.28 × 10^9^ CFU, which was 9.59 times as high as the value required by HPS2 strain, indicating that the virulence of ΔluxS strain significantly reduced, compared with that of HPS2 strain. The virulence of complemented mutant strain C-luxS (8.89 × 10^8^ CFU) was partially restored (Table 2).

**Table 2:** LD_50_ value of HPS2, ΔluxS and C-luxS strains in mice.

**Strains**	**Challenge dose (CFU)**	**Percent of dead mice (%)**	**Value of LD_**50**_ CFU**	Fold change^*^
HPS2	9.63 × 10^7^	0 (0/10)	4.46 × 10^8^	1
	2.08 × 10^8^	20 (2/10)		
	4.47 × 10^8^	30 (3/10)		
	9.63 × 10^8^	100 (10/10)		
ΔluxS	7.57 × 10^8^	0 (0/10)	4.28 × 10^9^	9.59
	1.55 × 10^9^	0 (0/10)		
	3.34 × 10^9^	20 (2/10)		
	7.57 × 10^9^	90 (9/10)		
C-luxS	5.83 × 10^8^	0 (0/10)	8.89 × 10^8^	1.99
	1.17 × 10^9^	90 (9/10)		
	2.25 × 10^9^	100 (10/10)		
	5.83 × 10^9^	100 (10/10)		

* *Fold change normalized to the wild-type strain (HPS2)*.

### The Tissue Burdens of HPS2, ΔluxS, and C-luxS Strains

**Figure 9:**
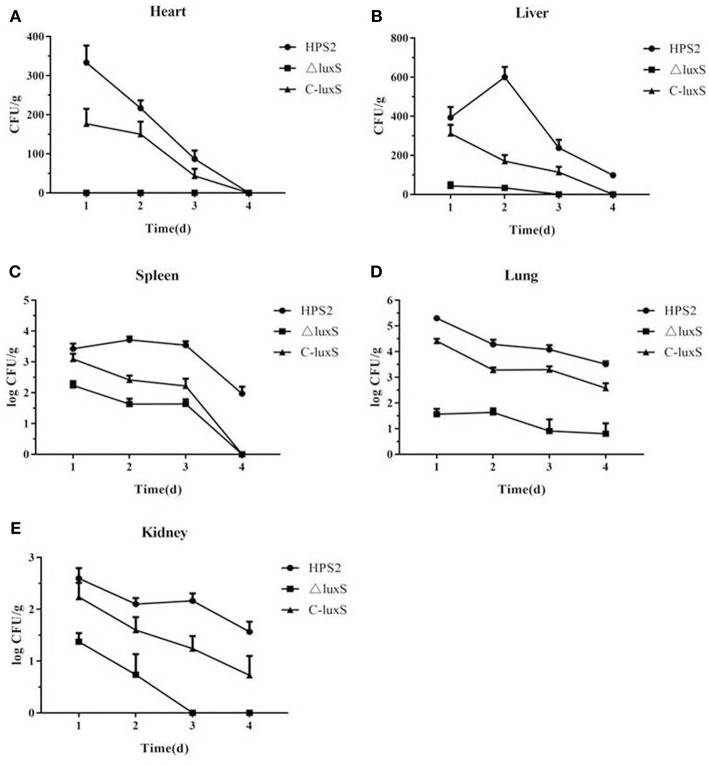
Bacterial distribution in heart **(A)**, liver **(B)**, spleen **(C)**, lung **(D)**, and kidney **(E)** of infected mice. The number of colonies was counted from day1 to day 4 post infection, and the results were expressed as CFU/1 g for all tissue samples. Error bars represent the standard deviation from three independent experiments performed in triplicate.

To further evaluate the virulence of HPS2, ΔluxS, and C-luxS strains, viable bacteria in infected mice organs were counted. As seen in Figure 9, bacteria were isolated from all heart, liver, spleen, lung and kidney tissues in three kinds of group mice. The number of bacteria in the lung was the largest, follow by spleen, kidney, liver, and heart, and the bacteria decreased gradually from the first day to the last day. Bacterial counts of ΔluxS-infected mice were significantly decreased compared with those of HPS2 or C-luxS-infected mice in all collected tissues (Figure 9), suggesting the colonization ability of HPS2 and C-luxS strains was obviously stronger than ΔluxS strain.

## Discussion

L*uxS* amino acid sequences were grouped into three major distinct clusters in the phylogenetic tree in the previous studies (Sun et al., 2004; Rao et al., 2016), and the *luxS* quorum sensing system was reported to be present in approximately half of all sequenced bacterial genomes (Waters and Bassler, 2004). Therefore, *luxS* gene could play an important identification role in species recognition to some extent. *LuxS*/AI-2 system was reported to exist in *A. actinomycetemcomitans, A. pleuropneumoniae, H. influenzae*, and *Mannheimia haemolytica* of the family of Pasteurellaceae. *LuxS* inactivation affected the physiological features and/or virulence of the above-mentioned strains (Fong et al., 2003; Daines et al., 2005; van der Vinne et al., 2005; James et al., 2006b; Li et al., 2008). In this study, *luxS* genes from the genomes of 15 kinds of standard reference strains of *H. parasuis* were identified and their homology was highly conserved, compared with that of *luxS* genes of *Pasteurella* strains, indicating that *luxS* gene in *H. parasuis* may play a similar role in other strains.

Mutant strain ΔluxS and complemented strain C-luxS were constructed and polarity effect also been verified to make sure that the change in function of ΔluxS strain was only attributed to *luxS* gene, rather than other upstream or downstream genes. This study found that the growth characteristics of the examined strains were similar except that the growth rate of C-luxS strain was about 2 h slower than that of HPS2 and ΔluxS strains, and that all strains exhibited a decrease in OD_600_ value after 18 (HPS2 and ΔluxS strains) or 20 (C-luxS strain) hours of culturation in T/V/S medium, rather than remained stable after reaching stationary phase, and which might be due to the dissolution of bacteria in nutrient deficient medium.

The results of AI-1 and AI-2 detection assay showed that *H. parasuis* only produced AI-2 molecule and that no AI-1 molecule could be detected in the supernatant of 15 kinds of standard reference strains of *H. parasuis*. In addition, when *luxS* gene was deleted from HPS2, the luminescence value was almost the same with that detected in the negative group (Figure 2C). Based on it, it could be inferred that *luxS*/AI-2 might be probably the only kind of quorum sensing system existing in *H. parasuis*, and that AI-2 might also be a kind of common molecules in all *H. parasuis* strains. But the expression levels of different serotype strains were different and could not reach the level expressed by the reporter strain *V. harveyi* BB170 (Figures 2B, C), which might be due to the fact that the structure of AI-2 was different in various species to some degree (Miller et al., 2004). As seen in Figure 2A, the amount of AI-2 molecule reached its maximum in early stationary phase, and it decreased sharply after stationary phase (Figure 2A). The AI-2 molecule variation pattern of HPS2 was similar to that of *M. haemolytica* A1 (Malott and Lo, 2002), *C. perfringens* (Ohtani et al., 2002), and *Streptococcus* mutant (Merritt et al., 2003). However, in many other species, such as *A. pleuropneumoniae* (Li et al., 2008), *A. actinomycetemcomitans* (Fong et al., 2001), enterohemorrhagic and enteropathogenic *E. coli* (Bowden and Li, 1997) and *Porphyromonas gingivalis* (James et al., 2006a), the amount of AI-2 molecule reached the maximum in exponential phase, suggesting that the function of AI-2 signal molecule might vary with different species.

In some strains, such as *A. pleuropneumoniae, Streptococcus mutants*, and *P. gingivalis, luxS* gene was verified to be associated with stress response in previous studies (Wen and Burne, 2004; Yuan et al., 2005; Li et al., 2008; Ma et al., 2017). In the heat shock assay, we found ΔluxS strain showed weak resistance to high temperature (48°C), which was consistent with another finding of our study that the growth activity of ΔluxS strain was inhibited under 40°C. In addition, our study revealed that the ability of ΔluxS strain against oxidative stress decreased, which agreed with the previous study results of ΔluxS strain of *A. pleuropneumoniae* (Li et al., 2008). Whereas, our result was in contrast with the previous study of ΔluxS strain of *Streptococcus mutans* (Wen and Burne, 2004) and *P. gingivalis* (Yuan et al., 2005). To explore the reasons why significant differences in growth characteristic and the ability to tolerate stress resistance were observed between HPS2 and ΔluxS, the expression level of some transcriptional regulatory genes was quantified, based on the reports that *htrA* is a kind of very important heat shock induced serine protease (Pallen and Wren, 2010), and that the growth characteristic of its mutant strain is inhibited at high temperature (Zhang et al., 2016). The quantitative results showed that the amount of mRNA of *htrA* gene in ΔluxS strain was obviously lower than that of HPS2 and C-luxS strains, which may explained why ΔluxS strain displayed the deficient growth characteristic at high temperature and weak resistance to stress response (Supplementary Figure 2).

The wild-type standard reference strain HPS2 was reported to have weak ability to form biofilm (Jin et al., 2006). In this study, a small amount of biofilm was observed, when HPS2 was cultured in T/V/S medium over 24 h, which may due to the difference of culture conditions (Figure 5). As reported in previous study, the vast majority of bacteria in natural and clinical environments exist in form of biofilm and not as the free-living or “planktonic” cells (Kaplan and Mulks, 2005). Biofilm also plays a key role in the pathogenesis of many bacterial infections (Parsek and Singh, 2003). In this study, AI-2 molecular synthesized by *luxS* gene was found to have inhibited the biofilm formation. which was consistent with the previous studies of *A. pleuropneumoniae* (Li et al., 2008, 2011) and *H. influenzae* (Daines et al., 2005; Armbruster et al., 2009, 2011; Pang et al., 2018). But other previous studies reported that AI-2 molecular could enhance the ability of biofilm formation in some bacteria, such as *S. suis* (Merritt et al., 2003), *Staphylococcus aureus* (Yu et al., 2012), and *Pseudomonas aeruginosa* (Sakuragi and Kolter, 2007), It may be attributed to the complexity of biofilm formation involving the multifactors which participated in adherence, metabolism, quorum sensing, and the stress response, and other processes (Hasona et al., 2007). On the other hand, *htrA* was found to have inhibited the formation of biofilm in *H. parasuis* (Zhang et al., 2016). Meanwhile, our quantitative results of *htrA* in HPS2, ΔluxS, and C-luxS strains supported the result of previous study.

It has been reported that the abilities of autoagglutination, hemagglutination, and adherence were virulence-associated markers (Janda et al., 1987; Fitzgerald et al., 1999; Liu et al., 2016). The adherence of bacteria to host cell surfaces was an essential determinant for bacterial colonization and cellular invasion which contributed to breaching the cell barriers, persistent infection in the host, ultimately resulting in systemic disease (Vahle et al., 1997). This study found that the abilities of autoagglutination, hemagglutination, and adherence of ΔluxS strain were sharply decreased, compared with those of HPS2 strain, which, in turn, proved the decrease of virulence in ΔluxS strain. Similar results were also found in *C. jejuni* strain. The autoagglutination ability of *luxS* mutant decreased, compared with that of wild-type strain (Jeon et al., 2003). Meanwhile, the variation pattern of hemagglutination and adherence abilities in ΔluxS strain was found to be the same with that of *Haemophilus ducreyi* and *Actinobacillus pleuropneumoniae*, respectively (Labandeirarey et al., 2010; Liu et al., 2016). However, the growth characteristic at 40°C and abilities of autoagglutination, hemagglutination, and adherence could not be absolutely rescued in complemented strain C-luxS, which may be due to the difference of transcriptional level of *luxS* gene in wild-type and complemented strains, in turn, its transcriptional level of related genes would also be changed. So, compared with wild-type strain, the balance of regulatory network in complemented strain was changed. On the other hand, the detailed relationship between autoagglutination, hemagglutination, and adherence remains unclear. Therefore, their complicated regulatory network remains to be further investigated.

*LuxS* gene was previously identified as a virulence determinant contributing to intracellular survival in *A. pleuropneumoniae* (Li et al., 2008), *H. influenzae* (Daines et al., 2005), and *Streptococcus* (Wen and Burne, 2004; Ma et al., 2017). To further evaluate the role of *luxS* gene in the pathogenesis of *H. parasuis in vivo*, LD_50_ and tissue burdens of bacteria in mice organs were measured. LD_50_ experiment showed that the virulence of ΔluxS strain attenuated about 10 times. The ΔluxS strains were found to have sharply decreased in heart, liver, spleen, lung, and kidney, compared with HPS2. Lung is the major target organ for *H. parasuis* to reside. The largest amount of bacteria were separated from it. As the time went on, the total amount of HPS2, ΔluxS, and C-luxS strains in organs were obviously declined in the tissues, suggesting that the colonization ability and virulence of ΔluxS strain decreased.

## Conclusions

In summary, we constructed the *luxS* deletion mutant and its complemented strain from *H. parasuis* serovar 2, and preliminarily investigated the effects of *luxS* gene on several virulence-associated properties. The comparison of ΔluxS with HPS2 and C-luxS strains revealed that *luxS* gene was related to phenotypic characteristics and biological abilities including growth characteristic, stress response, quorum sensing, biofilm formation, autoagglutination, hemagglutination, adherence, and virulence-associated LD_50_ and tissue burdens of bacteria. This study provides an insight into the role of the *luxS* gene in the pathogenesis of *H. parasuis* infection.

## Author Contributions

BZ and QH: conceptualization and writing—review & editing; BZ and XK: data curation; BZ, XZ, and YZ: formal analysis; QH: funding acquisition; BZ, XK, and YZ: investigation; BZ, XK, YZ, and QH: methodology; QH: project administration; FC, XZ, and GC: software; QH: supervision; BZ, GC, WZ, JL, and LZ: visualization; BZ: writing—original draft.

### Conflict of Interest Statement

The authors declare that the research was conducted in the absence of any commercial or financial relationships that could be construed as a potential conflict of interest.
