# Supplementary material for: The AI-2/luxS Quorum Sensing System Affects the Growth Characteristics, Biofilm Formation, and Virulence of Haemophilus parasuis
Source: Front Cell Infect Microbiol. 2019 Mar 19;9:62. doi: 10.3389/fcimb.2019.00062 (PMC6434701; doi:10.3389/fcimb.2019.00062)
Supplement: Supplementary file 1 [file Data_Sheet_1.docx]

Supplementary data:

**
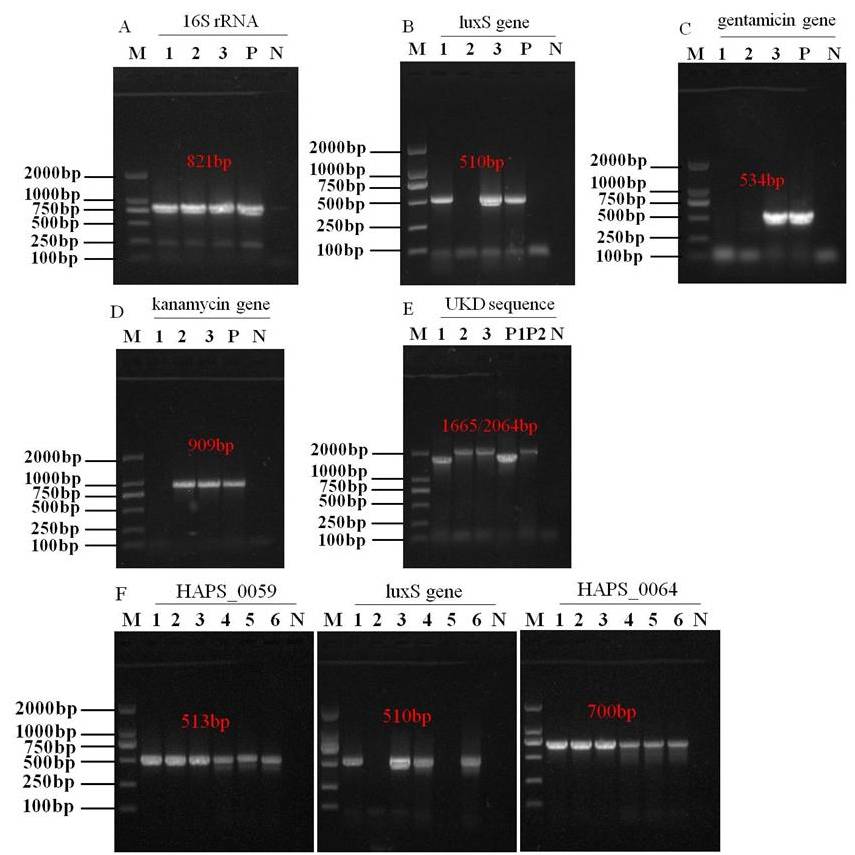
**

**Figure 1.** Construction and verification of *luxS* mutant strain and the complemented strain. (A-E) Identification results of the wild-type HPS2, deletion mutant ΔluxS and complemented strain C-luxS. The 16S rRNA, *luxS* gene, gentamicin gene, kanamycin resistance cassette sequence and UKD sequence were amplified, respectively. 1 to 3 represent genomes of the HPS2, ΔluxS and C-luxS strains, respectively, P: positive control, N: negative control. (F) RT-PCR identification of the HAPS_0059, *luxS* gene and HAPS_0064 from cDNAs of the HPS2, ΔluxS, and C-luxS strains. Amplification with the genomic DNA (gDNA) of the strains was used as the control. 1 to 3 represented cDNA genomes of the HPS2, ΔluxS and C-luxS strains, respectively, 4 to 6 represented genomes DNA (gDNA) of the HPS2, ΔluxS and C-luxS strains, respectively, which were positive controls, N: negative control.


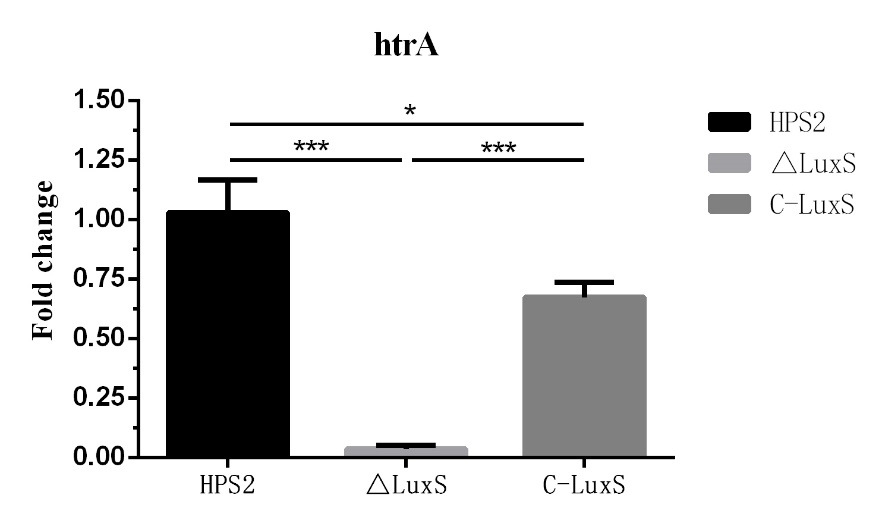


**Figure 2.** Quantitative results of *htrA* gene in HPS2, ΔluxS, and C-luxS strains. The assay was performed in triplicate for three times. Bars represent the mean ± standard deviation of three independent experiments. Statistical analyses were performed using the two-way ANOVA. * p<0.05 and *** p<0.001 represent increasing degrees of significant differences, respectively.

**Table 1. The nucleotide and protein homology of *luxS* genes between *H. parasuis* with several different *Pasteuriaceae* strains.**

| Strain | Strain | Nucleotide homology (%) | Protein homology (%) |
| --- | --- | --- | --- |
| *H. parasuis* | *A. pleuropneumoniae* | 78 | 83 |
|  | *Pasteurella multocida* | 69 | 73 |
|  | *H. influenzae* | 71 | 74 |

Sequence results：

1. Promotor and *luxS* gene sequence

ATACAGAATTTTGATTGAACTGACTAGAAAGCAAGCGGTTAGATTGGCGTAAAAATTTGCAAATTATTTCGAATAACTTACCGCTTGTATGAAAACTCAGGCTTAAATAGGCTATAATCCCTACATAATTTTATTTATTGGAGAACTGTTATGCCTTTACTAGATAGCTTTAAAGTTGACCACACCAAAATGAATGCCCCAGCAGTGCGTGTTGCAAAAACCATGACAACCCCGAAGGGCAATACGATTACTGTATTTGATTTACGTTTTGTCCGTCCAAACATTGAGATTTTATCGCCTCGTGGTATTCATACCATGGAACATTTATTTGCTGGTTTTATGCGTGATCATCTCAATAGCGATACCGTTGAAATTATTGATATTTCCCCGATGGGTTGCCGTACGGGATTTTATATGTCGTTAATTGGTTCACCTTCGGCTGAAGAAGTGGCAAAAGCGTGGGAAGCCTCTATGCGTGATGCGTTAGAAAAAGTGCCTGATGAGTCTAAAATTCCTGAATTAAACGAATATCAATGTGGCTCTTATAAAGAACACTCTTTAGCTGATGCACACGAAATTGCGCGTAATGTGTTAAAACAACCGATTGGTATTAACCGTAACGAAGATTTAGCATTAGATGAGAAATTGCTAAATCCATAG

2. UKD sequence (*luxS* upstream sequence, kanamycin resistance cassette sequence and *luxS* downstream sequence)

ATGCTTACGTCCTGATAAAGCTCATACTTTGTCCGAGCATCAAGCGGTGGAAATTCCAACGCAACCGCATCGGAAATACCCCTTGTAACGACTGAAAGCGAACAGCACAGCCGAGATAGACAAACACCACACTCATCAATGCCACAATCAGCATTAATATCAGAGAAGTGCCTTTTTCTTGCCACAACTGTTGAGCAGAATAAGTGTTTACCCAGTCCACCAACTCGCCCATATACTGAAACAAAATCCCTTCAATAATGCCTATAAATGCAACAAAAATGATTAACGCCACAAAATAGCTTCTCATTCCTTTGGTACTTTCAAAAATAAAAGGAATAACCTTTGCTCGAGGCGTATTCGGCATATCTTCTGGATAAGGATTAATGCGATTTTCAAACCAATCAAATAATTTATTTAACACGGGCTCTCCTTATAAGTATGGTGGGTAAAAATTGCAAGGTAATAAAATGTAGTGGACACACTCAGTGTGCCCCCTACCATTTGAAAAGAAACTATTGTTAGTTCCGTAGCAATAACAATTAACCAATTCTGATTAGAAAAACTCATCGAGCATCAAATGAAACTGCAATTTATTCATATCAGGATTATCAATACCATATTTTTGAAAAAGCCGTTTCTGTAATGAAGGAGAAAACTCACCGAGGCAGTTCCATAGGATGGCAAGATCCTGGTATCGGTCTGCGATTCCGACTCGTCCAACATCAATACAACCTATTAATTTCCCCTCGTCAAAAATAAGGTTATCAAGTGAGAAATCACCATGAGTGACGACTGAATCCGGTGAGAATGGCAAAAGCTTATGCATTTCTTTCCAGACTTGTTCAACAGGCCAGCCATTACGCTCGTCATCAAAATCACTCGCATCAACCAAACCGTTATTCATTCGTGATTGCGCCTGAGCGAGACGAAATACGCGATCGCTGTTAAAAGGACAATTACAAACAGGAATCGAATGCAACCGGCGCAGGAACACTGCCAGCGCATCAACAATATTTTCACCTGAATCAGGATATTCTTCTAATACCTGGAATGCTGTTTTCCCGGGGATCGCAGTGGTGAGTAACCATGCATCATCAGGAGTACGGATAAAATGCTTGATGGTCGGAAGAGGCATAAATTCCGTCAGCCAGTTTAGTCTGACCATCTCATCTGTAACATCATTGGCAACGCTACCTTTGCCATGTTTCAGAAACAACTCTGGCGCATCGGGCTTCCCATACAATCGATAGATTGTCGCACCTGATTGCCCGACATTATCGCGGGCCCATTTATACCCATATAAATCAGCATCCATGTTGGAATTTAATCGCGGCCTCGAGCAAGACGTTTCCCGTTGAATATGGCTCATAACACCCCTTGTATTACTGTTTATGTAAGCAGACAGTTTTATTGTTCATGATGATATATTTTTATCTTGTGCAATGAACGATTCTCCAATAAATAAAATTATGTAGGGATTATAGCCTATTTAAGCCTGAGTTTTCATACAAGCGGTAAGTTATTCGAAATAATTTGCAAATTTTTACGCCAATCTAACCGCTTGCTTTCTAGTCAGTTTGGTGAAAATTCTGTATAATCGCCCGTCTTTTGTGATCTTCATCACAATTTTGGATTTCGTCAGATGAGCTTTAGCTCATCATTTTTTATTCTCGGTGGGCGAGCCCCACCCTACAATTAAGTTATTTCAATGCAACAATTAGATACTCAAAAACTGCGTAATATCGCAATCATTGCTCACGTTGACCACGGCAAAACCACGCTGGTTGATAAATTATTAAAACTTTCAGGCACATTAGACACTTCTCGTGGTGATGTAGATGAACGTGTAATGGACTCCAACGACCTTGAGAAAGAGCGTGGCATTACCATTCTTGCGAAAAATACCGCAATTAACTGGAACGGCTATCGTATCAATATCGTAGATACCCCAGGACACGCAGACTTCGGTGGTTAAAGTGAAGCCGG

**Table 2. The nucleotide homology of *luxS* genes between different serotype of *H. parasuis* strains.**


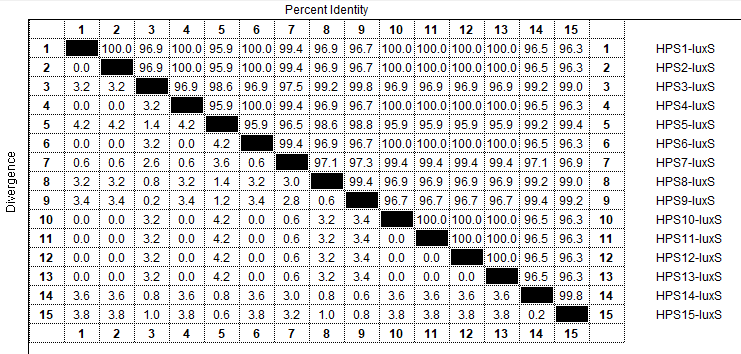


The detail sequences of *luxS* gene in different reference strains:

>Actinobacillus pleuropneumoniae L20 serotype 5b

ATGCCTTTATTAGATAGCTTTAAAGTGGATCACACTCGTATGAACGCACCGGCAGTGCGTGTTGCAAAAACGATGACGACGCCGAAGGGCGATACGATTACCGTATTCGATTTACGTTTTTGCCGTCCGAATATTGATATTTTACCGGTGCGTGGTATCCACACGATGGAACACTTATTTGCCGGCTTTATGCGTGATCATTTAAATAGTGAAAGTGTAGAAATTATTGATATTTCGCCGATGGGTTGCCGTACCGGTTTCTATATGTCGTTAATCGGTGCGCCAAGCGAAGCGGATGTGGTTTCGGCGTGGACAAAATCGATGGAAGATGCTTTAAATAAAGTACCGGACGTGTCAAAAATTCCGGAATTAAACGAATATCAATGCGGCTCTTATAAAGAGCATTCGCTTGAAGAAGCGCATCAAATCGCTCGTGATGTGTTAGCCAAGGGTATCGGCGTAAACCGTAACGAAGATTTAGCGCTTGATGAGAAATTATTAAATCCATAA

>Actinobacillus pleuropneumoniae serovar 3 str. JL03

ATGCCTTTATTAGATAGCTTTAAAGTGGATCACACTCGTATGAACGCGCCGGCAGTGCGTGTTGCAAAAACGATGACGACGCCGAAGGGCGATACGATTACCGTATTCGATTTACGTTTTTGCCGTCCGAATATTGATATTTTACCGGTGCGTGGTATCCACACGATGGAACACTTATTTGCCGGCTTTATGCGTGATCATTTAAATAGTGAAAGTGTAGAAATTATTGATATTTCGCCGATGGGTTGCCGTACCGGTTTCTATATGTCGTTAATCGGTGCGCCAAGCGAAGCGGATGTGGTTTCGGCGTGGACAAAATCGATGGAAGATGCTTTAAATAAAGTACCGGACGTGTCAAAAATTCCGGAATTAAACGAATATCAATGCGGCTCTTATAAAGAGCATTCGCTTGAAGAAGCGCATCAAATCGCTCGTGATGTGTTAGCCAAGGGTATCGGCGTAAACCGTAACGAAGATTTAGCGCTTGATGAGAAATTATTAAATCCATAA

>Actinobacillus pleuropneumoniae strain KL 16

ATGCCTTTATTAGATAGCTTTAAAGTGGATCACACTCGTATGAACGCACCGGCAGTGCGTGTTGCAAAAACGATGACGACGCCGAAGGGCGATACGATTACCGTATTCGATTTACGTTTTTGCCGTCCGAATATTGATATTTTACCGGTGCGTGGTATCCACACGATGGAACACTTATTTGCCGGCTTTATGCGTGATCATTTAAATAGTGAAAGTGTAGAAATTATTGATATTTCGCCGATGGGTTGCCGTACCGGTTTCTATATGTCGTTAATCGGTGCGCCAAGCGAAGCGGATGTGGTTTCGGCGTGGACAAAATCGATGGAAGATGCTTTAAATAAAGTACCGGACGTGTCAAAAATTCCGGAATTAAACGAATATCAATGCGGCTCTTATAAAGAGCATTCGCTTGAAGAAGCGCATCAAATCGCTCGTGATGTGTTAGCCAAGGGTATCGGCGTAAACCGTAACGAAGATTTAGCGCTTGATGAGAAATTATTAAATCCATAA

>Actinobacillus pleuropneumoniae serovar 8 MIDG2331

ATGCCTTTATTAGATAGCTTTAAAGTGGATCACACTCGTATGAACGCACCGGCAGTGCGTGTTGCAAAAACGATGACGACGCCGAAGGGCGATACGATTACCGTATTCGATTTACGTTTTTGCCGTCCGAATATTGATATTTTACCGGTGCGTGGTATCCACACGATGGAACACTTATTTGCCGGCTTTATGCGTGATCATTTAAATAGTGAAAGTGTAGAAATTATTGATATTTCGCCGATGGGTTGCCGTACCGGTTTCTATATGTCGTTAATCGGTGCGCCAAGCGAAGCGGATGTGGTTTCGGCGTGGACAAAATCGATGGAAGATGCTTTAAATAAAGTTCCGGACGTGTCAAAAATTCCGGAATTAAACGAATATCAATGCGGCTCTTATAAAGAGCATTCGCTTGAAGAAGCGCATCAAATCGCTCGTGATGTGTTAGCCAAGGGTATCGGCGTAAACCGTAACGAAGATTTAGCGCTTGATGAGAAATTATTAAATCCATAA

>Actinobacillus pleuropneumoniae serovar 7 str. AP76

ATGCCTTTATTAGATAGCTTTAAAGTGGATCACACTCGTATGAACGCACCGGCAGTGCGTGTTGCAAAAACGATGACGACGCCGAAGGGCGATACGATTACCGTATTCGATTTACGTTTTTGCCGTCCGAATATTGATATTTTACCGGTGCGTGGTATCCACACGATGGAACACTTATTTGCCGGCTTTATGCGTGATCATTTAAATAGTGAAAGTGTAGAAATTATTGATATTTCGCCGATGGGTTGCCGTACCGGTTTCTATATGTCGTTAATCGGTGCGCCAAGCGAAGCGGATGTGGTTTCGGCGTGGACAAAATCGATGGAAGATGCTTTAAATAAAGTACCGGACGTGTCAAAAATTCCGGAATTAAACGAATATCAATGCGGCTCTTATAAAGAGCATTCGCTTGAAGAAGCGCATCAAATCGCTCGTGATGTGTTAGCCAAGGGTATCGGCGTAAACCGTAACGAAGATTTAGCGCTTGATGAGAAATTATTAAATCCATAA

>Bacillus anthracis ames

ATGCCATCAGTAGAAAGCTTTGAATTAGATCATACGATTGTAAAGGCACCTTATGTAAGACATTGCGGAGTTCACAATGTAGGTAGTGACGGTATTGTAAATAAATTCGATATTCGTTTTTGCCAACCGAATAAACAAGCAATGAAACCAGATGTTATTCATACGTTAGAACATTTATTAGCATTTAATTTACGTAAATATATTGATCGTTATCCACATTTTGATATTATCGATATTTCACCAATGGGCTGCCAAACAGGATACTACCTTGTAGTAAGCGGAACACCGACAGTTCGAGAAATCATTGATTTATTAGAATTAACATTAAAAGATGCGGTTCAAATTACAGAAATTCCAGCTGCAAATGAAACACAATGTGGTCAAGCGAAGCTTCACGATTTAGAAGGAGCAAAACGCTTAATGAACTTCTGGTTAAGCCAAGATAAAGATGAACTTGAGAAAGTATTTGGATTG

>Bacillus cereus NC7401

ATGCCATCAGTAGAAAGCTTTGAATTAGATCATACGATTGTAAAGGCACCTTATGTAAGACATTGCGGAGTTCACAATGTAGGTAGTGACGGTATTGTAAATAAATTTGATATTCGTTTTTGCCAACCGAATAAACAAGCGATGAAACCAGATGTTATTCATACGTTAGAACATTTATTAGCATTTAATTTACGTAAATATATTGATCGTTATCCGCATTTCGATATTATCGATATTTCACCAATGGGCTGCCAAACAGGATACTACCTTGTAGTAAGCGGAACACCGACAGTTCGAGAAATCATTGATTTATTAGAATTAACATTAAAAGATGCGGTTCAAATTACAGAAATTCCAGCTGCAAATGAAACGCAATGTGGTCAAGCGAAGCTTCACGATTTAGAAGGAGCAAAACGCTTAATGAACTTCTGGTTAAGCCAAGATAAAGATGAACTTGAGAAAGTATTTGGATAG

>Bibersteinia trehalosi USDA-ARS-USMARC-190

ATGCCTTTATTAGATAGTTTTAAAGTTGATCATACTAAAATGCATGCCCCTGCTGTGCGTGTTGCTAAAACGATGACCACGCCAAAAGGCGATATTATTACGGTTTACGATCTTCGTTTTGTTCGTCCAAATGTGGAAATTCTTTCTCCTCGTGGCATTCACACCATGGAACATTTATTTGCTGGTTTTATGCGTGATCATTTAAATGCAGACGATGTAGAAATTATCGATATTTCGCCAATGGGGTGCCGTACTGGGTTCTATATGTCGTTAATCGGCTCACCATCAGCAGAACGAGTGGCTGATGCGTGGAAAAAATCGATGGAAGATGCACTAAATAAAGTGCCGGATGAAGCACATATTCCAGAACTCAATGAATACCAATGCGGTTCCTATAAAGAGCATTCTTTAGCCGATGCCCATGAAATTGCCCGTAACGTTCTAAAACAACCAATCGGCATTAACCACAATGAAGATTTAGCGTTAGATGAGAAATTGTTAAATCCATA

>Bifidobacterium longum strain BG7

ATGGCAGAAGAAACGGCCGAAAAGCCCGTCGTCGAATCATTCCAGCTTGATCACACCAAGGTCAAGGCGCCGTACGTGCTTACATCGACACCGAAACGGGCCCTCACGGCGACGTGATCTCCAACTATGATCTGCGCCTGACCCAGCCGAACAAGCAGGCCATCCCCACCGGCGGCCTGCACACCATCGAACACACCATCGCCGTGCTGCTGCGCGAACGTATCCCGGGCTACATCGACTGCTCGCCGTTCGGCTGCCGCACCGGCTTCCACCTGCTGACCTGGGGCACCCACCCCACCGAGGAAGTGGCCAAGGCCCTGAAGGAATCGTTGGAGTTCATCGCGTACAAGGCCACGTGGGATGACGTGCCCGCCACCACCGAGAAGAGCTGCGGCAACTATCGCGACCACAGCCTGTTCACCGCCAAGGAATGGGCCAAGCAGATCCTTGAGGAAGGCATCAGCTCCGACCCGTTCGAGCGCAAGGTCGTCTGA

>Borrelia burgdorferi B31

ATGAAAAAAATAACAAGCTTTACAATAGATCATACAAAACTCAACCCTGGCATATATGTCTCAAGAAAAGATACCTTTGAAAATGTAATATTTACTACAATAGACATTAGAATCAAAGCTCCCAACATCGAACCAATAATTGAAAACGCAGCAATACATACAATAGAGCACATAGGAGCTACTTTACTTAGAAATAATGAAGTTTGGACCGAAAAAATAGTATATTTTGGCCCTATGGGATGCAGAACTGGTTTTTACTTAATAATTTTTGGAGACTATGAAAGTAAAGATCTTGTTGACTTAGTCTCATGGCTTTTTTCCGAAATCGTAAATTTTTCAGAACCTATCCCAGGCGCAAGTGATAAGGAATGCGGAAATTACAAAGAACATAACCTTGATATGGCTAAATATGAATCTTCTAAATACTTACAAATATTAAACAATATTAAAGAAGAAAATTTAAAATATCCTTAG

>Campylobacter jejuni strain CJM1cam

ATGCCATTATTAGACAGCTTTAAAGTTGACCATACTAAAATGCCAGCTCCTGCTGTGCGTTTAGCTAAAGTTATGAAAACACCTAAGGGTGATGATATTAGCGTGTTTGATTTGCGTTTTTGTATACCAAATAAAGACATTATGAGCGAAAAAGGTACACATACCTTAGAACATTTATTTGCAGGATTTATGAGAGATCATCTTAATTCAAATTCAGTTGAAATTATTGATATTTCACCTATGGGTTGTCGCACGGGTTTTTATATGAGTTTAATTGGAACACCAGATGAAAAAAGTGTTGCAAAAGCTTGGGAAGAAGCTATGAAAGATGTTTTAAGCGTAAGCGATCAAAGCAAAATTCCTGAACTTAATATCTATCAATGCGGAACTTGCGCAATGCATTCTTTAGATGAAGCCAAACAAATTGCCCAAAAGGTTTTAAATCTAGGTATTAGCATAATGAATAACAAAGAATTAAAACTCGAGAATGCTTAA

>Campylobacter jejuni subsp. jejuni S3

ATGCCATTATTAGACAGCTTTAAAGTTGACCATACTAAAATGCCAGCTCCTGCTGTGCGTTTAGCTAAAGTTATGAAAACACCTAAGGGTGATGATATTAGCGTGTTTGATTTGCGTTTTTGCATACCAAATAAAGACATTATGAGCGAAAAAGGTACTCATACCTTAGAACATTTATTTGCAGGATTTATGAGAGATCATCTTAATTCAAATTCAGTTGAAATTATTGATATTTCACCTATGGGTTGTCGCACGGGTTTTTATATGAGTTTAATTGGAACACCAGATGAAAAAAGTGTTGCAAAAGCTTGGGAAGAAGCTATGAAAGATGTTTTAAGCGTAAGCGATCAAAGCAAAATTCCTGAACTTAATATCTATCAATGCGGAACTTGCGCAATGCATTCTTTAGATGAAGCCAAACAAATTGCCCAAAAGGTTTTAAATCTAGGTATTAGCATAATGAATAACAAAGAATTAAAACTCGAGAATGCTTAA

>Clostridium acetobutylicum DSM 1731

ATGGAAAAAATCGCAAGTTTTACTGTTAACCACTTAACACTTCAACCAGGTGTTTATGTTTCGAGAAAAGATAAATTTGGTGATGTAGTTATTACTACTTTTGATATACGAATGACAAGTCCAAATGAGGAACCAGTTATGAATACCGCTGAGGTTCATACTATAGAACATCTAGGGGCAACTTTTTTAAGAAATCACGGAACATATGCAGAAAAAACAGTATACTTTGGGCCAATGGGATGCAGAACAGGCTTTTACTTGATTTTACAGGGAGATTATACTTCAAATGATATTGTACCTTTATTAAGGGAAATGTATAAATTCATTGCAGATTTTAAAGGTGAGGTTCCAGGTGCTGCTGCACGTGATTGTGGAAATTATCTTGACATGAACCTACCTATGGCAAACTACTGGGGAAAGAAATTTTCAGCTCTCCTAGATAACATATCTGAAGATAGATTAAATTATCCAGAGTAA

>Enterococcus faecalis strain W11

ATGGCACGCGTAGAAAGTTTTGAATTAGATCACAACACAGTAAAAGCACCATATGTTCGCCTTGCTGGCACAGAACAAAATGGTGATGCGTTAGTCGAAAAATATGACTTACGTTTCTTACAACCAAACAAAGACGCCCTACCAACAGGCGCATTACACACGTTGGAACATTTATTAGCAGTTAACATGCGTGATGAATTAAAAGGAATCATTGACATTTCGCCAATGGGTTGCCGCACTGGTTTTTATATGATTATGTGGGATCAACATTCACCACAAGAAATCCGTGATGCATTAGTCAACGTTTTAAACAAAGTAATCAATACAGAAGTTGTTCCAGCAGTCTCTGCAAAAGAGTGTGGAAACTACAAAGATCATTCTTTATTTGCAGCGAAAGAATATGCAAAAATCGTCTTAGACCAAGGAATTAGTTTAGATCCATTTGAACGTATTCTGTAA

>Escherichia coli CFT073

ATGCCGTTGTTAGATAGCTTCACAGTCGATCATACCCGGATGGAAGCGCCTGCAGTTCGGGTGGCGAAAACAATGAACACCCCGCATGGCGACGCAATCACCGTGTTCGATCTGCGCTTCTGCGTGCCGAACAAAGAAGTGATGCCAGAAAGAGGGATCCATACCCTGGAGCACTTGTTTGCTGGTTTTATGCGTAACCATCTTAACGGTAATGGCGTAGAGATTATCGATATCTCGCCAATGGGCTGCCGCACCGGTTTTTATATGAGTCTGATTGGTACGCCAGATGAGCAGCGTGTCGCTGATGCCTGGAAAGCGGCAATGGAAGACGTGCTGAAAGTGCAGGATCAGAATCAGATCCCGGAACTGAACGTCTACCAGTGTGGCACTTACCAGATGCACTCGTTGCAGGAAGCGCAGGATATTGCGCGTAACATTCTGGAACGTGACGTGCGCATCAACAGCAACGAAGAACTGGCGCTGCCGAAAGAGAAGTTGCAGGAACTGCACATCTAG

>Escherichia coli str. K-12 substr. MC4100

ATGCCGTTGTTAGATAGCTTCACAGTCGATCATACCCGGATGGAAGCGCCTGCAGTTCGGGTGGCGAAAACAATGAACACCCCGCATGGCGACGCAATCACCGTGTTCGATCTGCGCTTCTGCGTGCCGAACAAAGAAGTGATGCCAGAAAGAGGGATCCATACCCTGGAGCACCTGTTTGCTGGTTTTATGCGTAACCATCTTAACGGTAATGGTGTAGAGATTATCGATATCTCGCCAATGGGCTGCCGCACCGGTTTTTATATGAGTCTGATTGGTACGCCAGATGAGCAGCGTGTTGCTGATGCCTGGAAAGCGGCAATGGAAGACGTGCTGAAAGTGCAGGATCAGAATCAGATCCCGGAACTGAACGTCTACCAGTGTGGCACTTACCAGATGCACTCGTTGCAGGAAGCGCAGGATATTGCGCGTAGCATTCTGGAACGTGACGTACGCATCAACAGCAACGAAGAACTGGCACTGCCGAAAGAGAAGTTGCAGGAACTGCACATCTAG

>Haemophilus influenzae strain 723

ATGCCATTACTTGATAGTTTTAAAGTGGATCACACAAAAATGAACGCACCTGCAGTACGCATTGCAAAAACGATGCGCACGCCAAAAGGCGATAATATTACTGTTTTTGATTTACGTTTTTGTATTCCAAACAAAGAAATTCTTTCCCCAAAAGGCATTCATACACTTGAACATTTATTTGCTGGATTTATGCGCGATCACTTAAATGGCGATAGCATAGAAATTATTGATATTTCTCCGATGGGATGTCGCACGGGATTTTATATGTCTTTGATTGGCACACCAAATGAACAGGAAGTGTCTGAGGCTTGGTTAGCTTCAATGCAAGATGTTTTAGGTGTACAAGATCAAGCTTCTATTCCCGAATTAAATATCTATCAATGCGGAAGCTATACGGAACATTCCTTAGAAGATGCACACGAAATTGCCAAAAATGTTATCGCACGCGGTATAGGTGTAAATAAAAATGAAGATTTGTCACTCGATAATTCCTTATTAAAATAG

>Haemophilus influenzae strain C486

ATGCCATTACTTGATAGTTTTAAAGTGGATCACACAAAAATGAACGCACCTGCAGTACGCATTGCAAAAACGATGCGCACGCCAAAAGGCGATAATATTACTGTTTTTGATTTACGTTTTTGTATTCCAAACAAAGAAATTCTTTCCCCAAAAGGCATTCATACACTTGAACATTTATTTGCTGGATTTATGCGCGATCACTTAAATGGCGATAGCATAGAAATTATTGATATTTCTCCGATGGGATGTCGCACGGGATTTTATATGTCTTTGATTGGCACACCAAATGAACAGGAAGTATCTGAGGCTTGGTTAGCTTCAATGCAAGATGTTTTAGGTGTACAAGATCAAGCTTCTATTCCCGAATTAAATATCTATCAATGCGGAAGCTATACGGAACATTCCTTAGAAGATGCACACGAAATTGCCAAAAATGTTATCGCACGCGGTATAGGTGTAAATAAAAATGAAGATTTGTCACTCGATAATTCCTTATTAAAATAG

>Haemophilus parasuis SH0165

ATGCCTTTACTAGATAGCTTTAAAGTGGATCACACTCGTATGAACGCTCCTGCGGTGCGTGTTGCAAAAACAATGACAACCCCGAAGGGAGATACGATTACTGTGTTTGATTTGCGTTTTGTCCGTCCAAACATTGAGATTTTATCACCTCGTGGTATTCATACGATGGAACATTTATTTGCTGGTTTTATGCGTGATCATCTCAATAGCGATACCGTTGAAATTATTGATATTTCCCCGATGGGTTGCCGTACGGGATTTTATATGTCGTTAATTGGTTCACCTTCAGCTGAAGAAGTGGCAAAAGCTTGGGAAGCCTCTATGCGTGATGCGTTAGAGAAAGTGCCTGATGAAACTAAAATTCCTGAGTTAAATGAGTTTCAATGTGGTTCATATAAGGAACATTCTCTTGCTGATGCACACGAAATTGTGCGTAATGTGTTAAAACAACCGATTGGTATTAACCGTAACAAAGATTTAGCATTAGATGAGAAATTGCTAAATCCATAG

>Haemophilus parasuis ZJ0906

ATGCCTTTACTAGATAGCTTTAAAGTGGATCACACTCGTATGAACGCTCCTGCGGTGCGTGTTGCAAAAACAATGACAACCCCGAAGGGAGATACGATTACTGTGTTTGATTTGCGTTTTGTCCGTCCAAACATTGAGATTTTATCACCTCGTGGTATTCATACGATGGAACATTTATTTGCTGGTTTTATGCGTGATCATCTCAATAGCGATACCGTTGAAATTATTGATATTTCCCCGATGGGTTGCCGTACGGGATTTTATATGTCATTAATTGGTTCACCTTCAGCTGAAGAAGTGGCAAAAGCTTGGGAAGCCTCTATGCGTGATGCGTTAGAGAAAGTGCCTGATGAAACTAAAATTCCTGAGTTAAATGAGTTTCAATGTGGTTCATATAAGGAACATTCTCTTGCTGATGCACACGAAATTGCGCGTAATGTGTTAAAACAACCGATTGGTATTAACCGTAACAAAGATTTAGCATTAGATGAGAAATTGCTAAATCCATAG

>Haemophilus] parasuis strain SH03

ATGCCTTTACTAGATAGCTTTAAAGTGGATCACACTCGTATGAACGCTCCTGCGGTGCGTGTTGCAAAAACAATGACAACCCCGAAGGGAGATACGATTACTGTGTTTGATTTGCGTTTTGTCCGTCCAAACATTGAAATTTTATCTCCACGTGGTATTCATACCATGGAACATTTATTTGCTGGTTTTATGCGTGATCATCTCAATAGCGATACCGTTGAAATTATTGATATTTCCCCGATGGGTTGCCGTACGGGATTTTATATGTCGTTAATTGGTTCACCTTCAGCTGAAGAAGTGGCAAAAGCTTGGGAAGCCTCTATGCGTGATGCGTTAGAGAAAGTGCCTGATGAAACTAAAATTCCTGAGTTAAATGAGTTTCAATGTGGTTCATATAAGGAACATTCTCTTGCTGATGCACACGAAATTGCGCGTAATGTGTTAAAACAACCGATTGGTATTAACCGTAACAAAGATTTAGCATTAGATGAGAAATTGCTAAATCCATAG

>Haemophilus] parasuis strain CL120103

ATGCCTTTACTAGATAGCTTTAAAGTGGATCACACTCGTATGAACGCTCCTGCGGTGCGTGTTGCAAAAACAATGACAACCCCGAAGGGCGATACGATTACTGTGTTTGATTTACGTTTTGTCCGTCCAAACATTGAGATTTTATCGCCTCGTGGTATTCATACGATGGAACATTTATTTGCTGGTTTTATGCGTGATCATCTCAATAGCGATACCGTTGAAATTATTGATATTTCGCCGATGGGTTGCCGTACGGGATTTTATATGTCGTTAATTGGTTCACCTTCGGCTGAAGAAGTGGCAAAAGCTTGGGAAGCCTCTATGCGTGATGCGTTAGAGAAAGTGCCTGATGAAACTAAAATTCCTGAGTTAAATGAGTTTCAATGTGGTTCATATAAGGAACATTCTCTTGCTGATGCACACGAAATTGTGCATAATGTGTTAAAACAACCGATTGGTATTAACCGTAACGAAGATTTA GCATTAGATGAGAAATTGCTAAATCCATAG

>Haemophilus] parasuis strain SC1401

ATGCCTTTACTAGATAGCTTTAAAGTGGATCACACTCGTATGAACGCTCCTGCGGTGCGTGTTGCAAAAACAATGACAACCCCGAAGGGCGATACGATTACTGTGTTTGATTTACGTTTTGTCCGTCCAAACATTGAGATTTTATCGCCTCGTGGTATTCATACGATGGAACATTTATTTGCTGGTTTTATGCGTGATCATCTCAATAGCGATACCGTTGAAATTATTGATATTTCGCCGATGGGTTGCCGTACGGGATTTTATATGTCGTTAATTGGTTCACCTTCGGCTGAAGAAGTGGCAAAAGCTTGGGAAGCCTCTATGCGTGATGCGTTAGAGAAAGTGCCTGATGAAACTAAAATTCCTGAGTTAAATGAGTTTCAATGTGGTTCATATAAGGAACATTCTCTTGCTGATGCACACGAAATTGTGCATAATGTGTTAAAACAACCGATTGGTATTAACCGTAACGAAGATTTAGCATTAGATGAGAAATTGCTAAATCCATAG

>Haemophilus] parasuis strain KL0318

ATGCCTTTACTAGATAGCTTTAAAGTGGATCACACTCGTATGAACGCTCCTGCGGTGCGTGTTGCAAAAACAATGACAACCCCGAAGGGAGATACGATTACTGTGTTTGATTTGCGTTTTGTCCGTCCAAACATTGAGATTTTATCACCTCGTGGTATTCATACGATGGAACATTTATTTGCTGGTTTTATGCGTGATCATCTCAATAGCGATACCGTTGAAATTATTGATATTTCCCCGATGGGTTGCCGTACGGGATTTTATATGTCGTTAATTGGTTCACCTTCGGCTGAAGAAGTGGCAAAAGCGTGGGAAGCCTCTATGCGTGATGCGTTAGAAAAAGTGCCTGATGAGTCTAAAATTCCTGAATTAAACGAATATCAATGTGGCTCTTATAAAGAACACTCTTTAGCTGATGCACACGAAATTGCGCGTAATGTGTTAAAACAACCGATTGGTATTAACCGTAACGAAGATTTAGCATTAGATGAGAAATTGCTAAATCCATAG

>Haemophilus pittmaniae strain NCTC13334

ATGCCATTGCTCGATAGTTTTAAAGTGGATCACACTCGTATGAACGCCCCTGCCGTGCGCGTTGCCAAGACTATGCGTACGCCAAAGGGCGATAACATCACCGTTTTCGACTTGCGTTTTTGCATTCCCAACAAAGAAATCCTACCACCTAAAGGAATTCATACCTTAGAGCATTTATTTGCAGGTTTTATGCGCGACCATTTAAATAATGATAGTGTAGAAATCATCGATATTTCGCCAATGGGCTGTCGTACAGGTTTTTACATGTCATTAATAGGTACGCCAAAGGAACAACAAGTCGCTGATGCATGGTTAGCCTCCATGAAAGATATTTTAACTGTACAAGATCAAAATCAAATTCCTGAACTAAATGAATACCAATGTGGTACTTACACCGAGCATTCTTTAGAAGAAGCACATGATATTGTTAAAAATGTGATTGCTCGCGGTGTGGGGATTAATAAAAATGACGATTTAGCACTTGATGAATCTTTCTTAAAATAA

>Helicobacter pylori 26695

ATGAAAACACCAAAAATGAATGTAGAGAGTTTTAATTTGGATCACACCAAAGTCAAAGCCCCTTATGTGCGTGTCGCTGATCGCAAAAAGGGCGTTAATGGGGATTTGATTGTCAAATACGATGTGCGCTTCAAGCAGCCCAACCAAGATCACATGGACATGCCTAGCCTACATTCTTTAGAGCATTTAGTCGCTGAAATTATCCGCAACCATGCCAGTTATGTCGTGGATTGGTCGCCTATGGGTTGCCAAACGGGATTTTATCTCACAGTGTTAAACCATGACAATTACACAGAGATTTTAGAGGTTTTAGAAAAGACCATGCAAGATGTGTTAAAGGCTACAGAAGTGCCTGCCAGCAATGAAAAGCAATGCGGTTGGGCGGCTAACCACACTTTAGAGGGTGCTAAGGATTTAGCGCGCGCTTTTTTAGACAAACGCGCTGAGTGGTCTGAAGTGGGGGTTTGA

>Lactobacillus plantarum strain KLDS1.0391

ATGGCTAAAGTAGAAAGTTTTACATTAGATCATACCAAGGTTTTAGCACCTTACGTTCGTAAAATTACGGTGGAAAATGGGCCTAAGGGTGATGCCATCACTAATTTTGATTTGCGGTTAGTTCAACCTAACAAGACCGCTATTGATACGGCGGGCTTACACACGATTGAACATATGTTGGCTGGGTTATTACGTGATCGTATGGATGGCGTGATTGACTGCTCACCATTTGGTTGTCGGACTGGTTTTCATTTGATTACCTGGGGTGAACATGACACCGTTGAAGTTGCTAAGGCATTGAAGTCCTCATTAGAATTCATTGCTGGCCCAGCTAAGTGGGAAGACGTACAAGGGACGACCATCGATAGCTGTGGGAATTACAAGGATCATTCTTTATTCTCAGCTAAGGAATGGGCCAAGTTGATTTTATCGCAAGGAATTTCATCGGATCCATTTGTACGCAAAGTCGTTGAATAG

>Lactobacillus plantarum strain SQ332

ATGGCTAAAGTAGAAAGTTTTACATTAGATCATACCAAGGTTTTAGCACCTTACGTTCGTAAAATTACGGTGGAAAATGGGCCTAAGGGTGATGCCATCACTAATTTTGATTTGCGGTTAGTTCAACCTAACAAGACCGCTATTGATACGGCGGGCTTACACACGATTGAACATATGTTGGCTGGGTTATTACGTGATCGTATGGATGGCGTGATTGACTGCTCACCATTTGGTTGTCGGACTGGTTTTCATTTGATTACCTGGGGTGAACATGACACCGTTGAAGTTGCTAAGGCATTGAAGTCCTCATTAGAATTCATTGCTGGCCCAGCTAAGTGGGAAGACGTACAAGGGACGACCATCGATAGCTGTGGGAATTACAAGGATCATTCTTTATTCTCAGCTAAGGAATGGGCCAAGTTGATTTTATCGCAAGGAATTTCATCGGATCCATTTGTACGCAAAGTCGTTGAATAG

>Lactococcus lactis NCDO 2118

ATGGCTGAAGTAGAATCTTTTCAATTAGACCACACAAAAGTACTTGCCCCTTATGTCCGTTTAATCGGTAGCGAAACTGGGCCTAAAGGCGATGTTATCACTAATTTTGACGTTCGTTTTGTTCAACCTAATGCGAATGCCATTGGAATGGCCGCTTTACACACTATTGAGCATAGCATGGCTAGTTTAATTCGCGATAGAATTGATGGCATGATTGACTTTTCACCTTTTGGATGTCAAACTGGTTTCCACATGATTATGTGGGGTGAACATAGTTCAGAAGAAATTGCTAAAGTAATTAAATCTTCTCTAGAAGAGCTTGCAAGTGACGAATTTGGTTGGGATAATGTCCCTGGTGTTGCTGAAAAAGAATGCGGAAATTACCGTAATCACTCACTTTTCGGTGCTAAAGAATGGTCTAAGAAAATTCTTTCTGAAGGAATTTCTACTGACCCTTACGAACGTAAAGTAATTTAA

>Listeria monocytogenes str.IZSAM_Lm_hs2008

ATGGCAGAAAAAATGAATGTAGAAAGTTTTAATTTGGACCATACGAAAGTAAAAGCACCTTTTGTGAGACTGGCGGGAACGAAAGTGGGCGTCCATGGAGATGAAATATACAAATATGATGTTCGCTTCAAACAACCCAATAAAGAACATATGGAAATGCCAGCGCTACACTCTTTAGAACATTTGATGGCAGAACTTGCAAGAAATCATACTGACAAATTAGTAGACATTAGTCCAATGGGATGCCAAACTGGCTTTTATGTTTCCTTCATTAATCATAGCGACTATGATGATGCACTAGAAATCATCGCCACAACACTAACGGATGTTTTAGCTGCGACAGAAGTTCCTGCATGCAATGAAGTCCAATGCGGTTGGGCAGCAAGCCATAGTTTAGAAGGTGCAAAAGCGCTTGCAGCAGAATTTTTAGACAAACGAGACGAATGGAAAAATGTATTTGGTGAATAA

>Mannheimia varigena USDA-ARS-USMARC-1296

ATGCCTTTATTAGATAGTTTTAAAGTAGATCATACTAAAATGAACGCCCCAGCAGTGCGTGTGGCAAAAACAATGACAACGCCAAAAGGCGATACGATTACTGTATTCGATTTACGTTTTTGTCGCCCGAATATTGATATTCTTCCTGCGCGTGGTATTCATACGATGGAACACCTTTTTGCAGGCTTTATGCGTGATCACCTAAACAGTGAAACAGTAGAAATCATTGATATTTCGCCAATGGGTTGTCGCACAGGTTTTTATATGTCGCTCATCGGCACACCATCTGCGCAAGAAGTGGCAGATGCATGGACAGCTTCTATGGAAGATGCGTTAAATAAAATCCCAGATGTATCAGCCATTCCAGAACTTAATGAATACCAATGCGGATCTTACAAAGAGCATTCATTAGAAGAAGCTCACAAAATTGCCCGTGATGTGTTAAGTGCTGGCATCGGCATTAACCGTAATGAAGATTTAGCATTAGACGAGAAATTATTAAATCCATAA

>Neisseria meningitidis strain B6116/77

ATGCCCCTACTAGACAGTTTCAAAGTCGATCACACCCGTATGCATGCCCCCGCCGTACGCGTGGCGAAAACCATGACTACGCCCAAAGGCGACACCATTACCGTGTTTGACCTGCGCTTTTGCGTTCCCAACAAAGAAATCCTGCCTGAAAAAGGCATACACACGCTGGAGCATTTGTTCGCCGGCTTTATGCGCGACCACTTGAACGGA

AACGGCGTGGAAATCATCGACATTTCCCCGATGGGCTGCCGCACCGGTTTCTACATGAGCCTTATCGGCACGCCTTCCGAACAGCAGGTCGCCGATGCGTGGCTGGCTTCGATGCAGGATGTTTTGAATGTCAAAGACCAAAGCAAAATCCCCGAGTTGAACGAATACCAATGCGGCACTTATCAAATGCACTCGCTCGCCGAAGCGCAGCAAATCGCGCAAAACGTGTTGGCGCGCAAAGTGGCGGTGAACAAAAACGAAGAGCTGACGCTGGATGAAGGGCTGCTGAACGCCTAA

>Neisseria meningitidis strain DE8555

ATGCCCCTACTAGACAGTTTCAAAGTCGATCACACCCGTATGCATGCCCCCGCCGTACGCGTGGCGAAAACCATGACCACGCCCAAAGGCGACACCATTACCGTGTTCGACCTGCGCTTTTGCATTCCCAACAAAGAAATCCTGCCTGAAAAAGGCATACACACGCTGGAGCATTTGTTCGCAGGTTTTATGCGCGACCACTTGAACGGCAACGGCGTGGAAATCATCGACATTTCCCCGATGGGCTGCCGCACCGGTTTCTACATGAGCCTTATCGGCACGCCTTCCGAACAGCAGGTCGCCGATGCATGGCTCGCCTCGATGCAGGATGTGGGCAATGTCAAAGACCAAAGCAAAATCCCCGAGTTGAACGAATACCAATGCGGCACTTATCAAATGCACTCGCTCGCCGAAGCGCAGCAAATCGCGCAAAACGTGTTGGCGCGCAAAGTGGCGGTGAACAAAAACGAAGAGCTGACGCTGGATGAAGGGCTGCTGAACGCCTAA

>Pasteurella multocida strain ATCC 43137

ATGCCATTACTTGATAGCTTTAAAGTTGACCATACCCGTATGAAAGCGCCAGCAGTACGCATTGCGAAAACCATGACTACCCCAAAGGGCGATAACATTACTGTATTTGATTTACGTTTTTGCATACCAAATAAAGAGATTCTTTCCCCAAAAGGGATTCATACTTTAGAACATCTTTTTGCGGGTTTTATGCGCGATCATTTAAATGGGACAGAAGTAGAAATTATTGATATTTCCCCGATGGGCTGTTGTACAGGGTTTTACATGTCATTAATTGGCACACCCAATGAGCAACAGGTGGCAGACGCTTGGTTAGCCTCAATGCGAGATGTGTTATTGGTAAAAGATCAAGCGCAGATCCCAGAATTAAATGCTTTCCAGTGCGGGACTTACACCGAGCACTCATTAGCCGAAGCACAACAAATCGCACACAATGTACTTGAGCGTGGTGTGAGTGTGAATAAAAATGAAGATTTATTACTCGACGAACAATTATTATCTCTTTAA

>Pasteurella multocida subsp. multocida str. HB03

ATGCCATTACTTGATAGCTTTAAAGTTGACCATACCCGTATGAAAGCGCCAGCAGTACGCATTGCGAAAACCATGACTACCCCAAAGGGCGATAACATTACTGTATTTGATTTACGTTTTTGCATACCAAATAAAGAGATTCTTTCCCCAAAAGGGATTCATACTTTAGAACATCTTTTTGCGGGTTTTATGCGCGATCATTTAAATGGGACAGAAGTAGAAATTATTGATATTTCCCCGATGGGCTGTTGTACAGGGTTTTACATGTCATTAATTGGCACACCCAATGAGCAACAGGTGGCAGACGCTTGGTTAGCCTCAATGCGAGATGTGTTATTGGTAAAAGATCAAGCGCAGATCCCAGAATTAAATGCTTTCCAGTGCGGGACTTACACCGAGCACTCATTAGCCGAAGCACAACAAATCGCACACAATGTACTTGAGCGTGGTGTGAGTGTGAATAAAAATGAAGATTTATTACTCGACGAACAATTATTATCTCTTTAA

>Salmonella enterica serovar Typhi str. CT18

ATGCCATTATTAGATAGCTTCGCAGTCGATCATACCCGGATGCAAGCGCCGGCGGTCCGGGGTGCAAAAACGATGAACACCCCGCATGGCGACGCAATCACCGTGTTTGATCTGCGTTTTTGCATTCCGAACAAAGAAGTGATGCCGGAAAAAGGGATTCATACGCTTGAGCATCTGTTTGCTGGCTTTATGCGCGACCACCTCAACGGTAACGGCGTTGAGATTATCGATATCTCGCCGATGGGCTGCCGCACCGGCTTTTACATGAGCCTGATTGGCACGCCGGACGAGCAGCGTGTTGCCGACGCTTGGAAAGCGGCGATGGCGGATGTGCTGAAAGTGCAGGATCAAAACCAGATCCCGGAGCTGAACGTTTACCAGTGCGGTACGTATCAGATGCACTCGCTCAGTGAAGCGCAGGACATTGCCCGTCATATTCTGGAGCGTGATGTGCGCGTGAACAGCAATAAAGAGCTGGCGCTGCCGAAAGAAAAACTGCAGGAATTGCATATTTAG

>Salmonella enterica serovar Typhi Ty2

ATGCCATTATTAGATAGCTTCGCAGTCGATCATACCCGGATGCAAGCGCCGGCGGTCCGGGTTGCAAAAACGATGAACACCCCGCATGGCGACGCAATCACCGTGTTTGATCTGCGTTTTTGCATTCCGAACAAAGAAGTGATGCCGGAAAAAGGGATTCATACGCTTGAGCATCTGTTTGCTGGCTTTATGCGCGACCACCTCAACGGTAACGGCGTTGAGATTATCGATATCTCGCCGATGGGCTGCCGCACCGGCTTTTACATGAGCCTGATTGGCACGCCGGACGAGCAGCGTGTTGCCGACGCTTGGAAAGCGGCGATGGCGGATGTGCTGAAAGTGCAGGATCAAAACCAGATCCCGGAGCTGAACGTTTACCAGTGCGGTACGTATCAGATGCACTCGCTCAGTGAAGCGCAGGACATTGCCCGTCATATTCTGGAGCGTGATGTGCGCGTGAACAGCAATAAAGAGCTGGCGCTGCCGAAAGAAAAACTGCAGGAATTGCATATTTAG

>Salmonella enterica serovar Typhimurium str. LT2

ATGCCATTATTAGATAGCTTCGCAGTCGATCATACCCGGATGCAAGCGCCGGCGGTCCGGGTTGCAAAAACGATGAACACCCCGCATGGCGACGCAATCACCGTGTTTGATCTGCGTTTTTGCATTCCGAACAAAGAAGTGATGCCGGAAAAAGGGATTCATACGCTTGAGCATCTGTTTGCTGGCTTTATGCGCGACCACCTCAACGGTAACGGCGTTGAGATTATCGATATCTCGCCGATGGGCTGCCGCACCGGCTTTTACATGAGCCTGATTGGCACGCCGGACGAGCAGCGTGTTGCCGACGCCTGGAAAGCGGCGATGGCGGATGTGCTGAAAGTGCAGGATCAAAACCAGATCCCGGAGCTGAACGTTTACCAGTGCGGTACGTATCAGATGCACTCGCTCAGTGAAGCGCAGGACATTGCCCGTCATATTCTGGAGCGTGATGTGCGCGTGAACAGCAATAAAGAGCTGGCGCTGCCGAAAGAAAAACTGCAGGAACTGCATATTTAG

>Shewanella oneidensis MR-1

ATGCCATTACTTGATAGCTTTACCGTTGACCATACTCGGATGAATGCACCTGCCGTGCGTGTTGCCAAACATATGACGACCCCAAAAGGCGATGCGATTACCGTATTCGATCTGCGTTTTTGCGCGCCAAATAAAGATATCCTGAGCGAGCGTGGCATCCACACTTTGGAACATTTGTTTGCGGGCTTTATGCGTGACCATTTAAATGGTGATGATGTGGAAATTATTGATATTTCTCCAATGGGCTGTCGCACTGGTTTTTATATGAGCCTGATTGGCGTGCCTACTGAACGTCAAGTAGCGGATGCGTGGCTTGCCTCAATGGAAGATGTACTGAAAGTTGTTGAACAATCTGAAATTCCTGAGCTGAACGAATATCAATGTGGCACCTATGAGATGCACTCTTTGGAGCAAGCGCAGGACATTGCACGTAATATTATCGCAGCTGGCGTGAGCGTTAACCGCAACGATGATTTAAAACTCAGTGATGAGATTCTAGGTAAACTCTAG

>Shigella flexneri 2002017

ATGCCGTTGTTAGATAGCTTCACAGTCGATCATACCCGGATGGAAGCGCCTGCAGTTCGGGTGGCGAAAACAATGAACACCCCGCATGGCGACGCAATCACCGTGTTCGATCTGCGCTTCTGCGTGCCGAACAAAGAAGTGATGCCAGAAAGAGGGATCCATACCCTGGAGCACCTGTTTGCTGGTTTTATGCGTAACCATCTTAACGGTAATGGTGTAGAGATTATCGATATCTCGCCAATGGGCTGCCGCACCGGTTTTTATATGAGTCTGATTGGTACGCCAGATGAGCAGCGTGTTGCTGATGCCTGGAAAGCGGCAATGGAAGACGTGCTGAAAGTGCAGGATCAGAATCAGATCCCGGAACTGAACGTCTACCAGTGTGGCACTTACCAGATGCACTCGTTGCAGGAAGCGCAGGATATTGCGCGTAGCATTCTGGAACGTGAGGTACGCATCAACAGCAACGAAGAACTGGCACTGCCGAAAGAGAAGTTGCAGGAACTGCACATCTAG

>Staphylococcus aureus strain: JP080

ATGACAAAAATGAATGTTGAAAGTTTCAATTTAGATCATACTAAAGTGGTTGCCCCATTTATTAGATTAGCGGGAACGATGGAAGGATTAAACGGAGATGTCATTCACAAATACGACATTCGTTTCAAACAACCAAACAAAGAACATATGGATATGCCAGGATTGCATTCATTAGAACATTTAATGGCTGAAAATATTAGAAATCATAGTGACAAAGTCGTTGATTTAAGTCCTATGGGTTGCCAAACTGGTTTCTATGTATCATTTATTAATCATGATAATTATGATGATGTATTAAATATTGTTGAAGCAACTTTAAATGATGTGCTAAATGCTACTGAAGTGCCAGCTTGTAATGAAGTACAATGTGGCTGGGCAGCTAGTCATTCATTAGAAGGTGCTAAAACTATCGCTCAAGCATTTCTAGACAAACGAAACGAATGGCATGATGTTTTCGGTACAGGAAAATAA

>Staphylococcus aureus strain MRSA252

ATGACAAAAATGAATGTTGAAAGTTTTAATTTAGATCATACTAAAGTGGTTGCCCCATTTATTAGATTAGCGGGAACGATGGAAGGATTAAACGGAGATGTCATTCACAAATACGACATTCGTTTCAAACAACCAAACAAAGAACATATGGATATGCCTGGATTGCATTCATTAGAACATTTAATGGCTGAAAATATTAGAAATCATAGTGACAAAGTCGTTGATTTAAGTCCTATGGGTTGCCAAACTGGTTTCTATGTATCATTTATTAATCATGATAATTATGATGATGTATTAAATATTGTTGAAGCAACTTTAAATGATGTGCTAAATGCTACTGAGGTGCCTGCTTGTAATGAAGTACAATGTGGCTGGGCAGCAAGTCATTCATTAGAAGGTGCTAAAACTATCGCTCAAGCATTTCTAGACAAACGAAACGAATGGCATGATGTTTTCGGTACAGGAAAATAA

>Staphylococcus carnosus TM300

ATGCCAAAAATGAACGTTGAAAGTTTCAATTTAGACCACACTAAAGTTGTTGCACCATTCGTACGTTTAGCAGGAACAGTAGAAGGCGCAAACGGCGATGTAATTAAAAAATATGATATTCGTTTCAAACAACCTAATAAAGAGCACATGAAAATGCCAGGATTGCATTCTTTAGAACATTTAATGGCTGAAAATATTAGAAACCATACTGATAAAGTGGTTGATTTAAGCCCTATGGGTTGCCAAACAGGATTTTATGTATCTTTAATCAATCATGATGATTATGAAGATGTTTTGAATATTATTGAAGCAACTTTAAAAGATGTTCTTGCAGCAGATGAAGTACCAGCATGCAACGAAGTTCAATGTGGTTGGGCAGCAAGTCACTCACTAGAAGGTGCGAAAGAAATTGCTCAAGATTTCTTAGACAAAAGAGATCAATGGGATAAAATTTTCAGTGAATAA

>Staphylococcus epidermidis strain SEI

ATGACTAAAATGAATGTAGAAAGCTTTAATTTAGACCATACTAAGGTTGTTGCACCTTTTATTCGTCTAGCCGGGACTATGGAAGGTCTTAATGGTGATGTCATACATAAATATGACATTCGTTTCAAACAGCCCAATAAGGAACATATGGATATGCCTGGTCTACATTCCTTAGAGCATTTAATGGCAGAAAACATTAGAAATCATACTGATAAAGTAGTAGATTTAAGTCCTATGGGTTGTCAAACTGGATTCTATGTTTCATTTATTAATCATGACGACTACGATGACGTATTAAATATTATCGATCAAACATTGCATGATGTGTTAAATGCTAGCGAAGTCCCAGCTTGTAATGAGGTTCAATGTGGTTGGGCTGCAAGTCATTCTTTAGAAGGTGCTAAAACAATTGCTCAAGCATTTTTAGATAAAAGAGAGCAATGGAATGACATCTACGGAGAAGGTAAATAA

>Streptococcus agalactiae strain GBS85147

ATGACAAAAGAAGTTGTCGTAGAGAGTTTTGAATTAGATCACACCATCGTTAAAGCACCCTACGTTCGTTTAATTTCAGAAGAAGTTGGACCGGTCGGAGACATCATTACTAACTTTGATATCCGCCTTATTCAGCCTAATGAAAATGCTATTGATACTGCTGGTTTACATACTATCGAGCACCTTCTGGCGAAGTTAATCCGCCAACGAATTAATGGTTTGATTGATTGTTCTCCTTTTGGTTGTAGAACTGGTTTTCACATGATTATGTGGGGAAAACAGGATGCTACTGAAATTGCTAAAGTCATCAAATCTAGTCTAGAGGCTATTGCTGGTGGGGTCACTTGGGAAGACGTTCCTGGAACAACCATTGAATCTTGTGGAAATTATAAAGACCACAGTCTCCACTCTGCTCAAGAATGGGCGAAATTAATTCTTTCCCAAGGGATTTCTGACAATGCTTTTGAGCGCCACATTGTCTGA

>Streptococcus agalactiae strain SS1

ATGACAAAAGAAGTTGTCGTAGAGAGTTTTGAATTAGATCACACCATCGTTAAAGCACCCTACGTTCGTTTAATTTCAGAAGAAGTTGGACCGGTCGGAGACATCATTACTAACTTTGATATCCGCCTTATTCAGCCTAATGAAAATGCTATTGATACTGCTGGTTTACATACTATCGAGCACCTTCTGGCGAAGTTAATCCGCCAACGAATTAATGGTTTGATTGATTGTTCTCCTTTTGGTTGTAGAACTGGTTTTCACATGATTATGTGGGGAAAACAGGATGCTACTGAAATTGCTAAAGTCATCAAATCTAGTCTAGAGGCTATTGCTGGTGGGGTCACTTGGGAAGACGTTCCTGGAACAACCATTGAATCTTGTGGAAATTATAAAGACCACAGTCTCCACTCTGCTCAAGAATGGGCGAAATTAATTCTTTCCCAAGGGATTTCTGACAATGCTTTTGAGCGCCACATTGTCTGA

>Streptococcus mutans UA159

ATGACAAAAGAAGTTACTGTTGAAAGCTTTGAACTTGATCACATTGCTGTAAAAGCCCCTTATGTCCGTCTTATTTCAGAAGAGTTTGGACCTAAAGGCGATCTTATTACCAATTTTGATATTCGCTTAGTACAGCCTAATGAAGACTCTATTCCGACTGCAGGCCTTCATACTATTGAACATTTACTGGCTAAGCTGATTCGTCAGCGTATTGACGGGATGATTGACTGTTCCCCTTTTGGCTGTCGTACTGGTTTTCATCTCATCATGTGGGGTAAGCATACAACAACCCAAATAGCCACAGTCATCAAAGCAAGTTTAGAAGAAATTGCTAATACAATCTCATGGAAAGATGTCCCTGGAACAACTATTGAGTCCTGTGGGAATTACAAAGATCATAGCCTTTTTTCAGCTAAAGAATGGGCAAAGCTGATTTTAAAACAAGGCATTTCAGATGATCCTTTTGAGCGTCATCTAGTGTAA

>Streptococcus pneumoniae strain 05-447

ATGTCAAAAGAAGTTATTGTCGAAAGTTTTGAACTTGACCACACCATTGTCAAAGCACCCTATGTTCGCTTGATTGGGGAAGAAACAGGACCAAAAGGAGACATCATCTCCAATTATGATATTCGCTTGGTGCAACCAAACGAAGACTCTATCCCTACTGCCGGCCTTCACACTATCGAGCACCTCTTAGCCAAACTCATCCGTACCCGC

ATTGACGGCATGATTGACTGTTCACCATTTGGTTGCCGCACAGGCTTCCACATGATTATGTGGGGACGTCACACCAGTGCTAAAATCGCAGCTGTTATCAAGGATTCGCTCAAGGAAATCGCTGAAACTACTACTTGGGAAGATGTCCCAGGGACAACCATCGAATCTTGCGGAAACTACAAGGACCACAGCCTCTTCTCTGCTAAAGAATGGGCAAAACTCATCTTGGAACAAGGGATTTCAGATGATGCCTTTGAACGTCATGTGATTTAA

>Streptococcus pneumoniae strain K15-115

ATGTCAAAAGAAGTTATTGTCGAAAGTTTTGAACTTGACCACACCATTGTCAAAGCACCCTATGTTCGCTTGATTGGGGAAGAAACAGGACCAAAAGGAGACATCATCTCCAATTATGATATTCGCTTGGTGCAACCAAACGAAGACTCTATCCCTACTGCCGGCCTTCACACTATCGAGCACCTCTTAGCCAAACTCATCCGTACCCGCATTGACGGCATGATTGACTGTTCACCATTTGGTTGCCGCACAGGCTTCCACATGATTATGTGGGGACGTCACACCAGTGCTAAAATCGCAGCTGTTATCAAGGATTCGCTCAAGGAAATCGCTGAAACTACTACTTGGGAAGATGTCCCAGGGACAACCATCGAATCTTGCGGAAACTACAAGGACCACAGCCTCTTCTCTGCTAAAGAATGGGCAAAACTCATCTTGGAACAAGGGATTTCAGATGATGCCTTTGAACGTCATGTGATTTAA

>Streptococcus pyogenes M1 GAS

ATGACAAAAGAAGTTATCGTCGAAAGTTTTGAGCTAGATCATACTATTGTAAAGGCCCCTTATGTTCGTCTTATTTCTGAAGAATTTGGACCCAAGGGCGATCGTATTACAAATTTTGATGTTCGCCTAGTGCAGCCTAACCAAAATTCTATTGAAACAGCCGGTTTGCATACCATTGAACACTTACTTGCCAAGCTCATCCGCCAACGCATTGATGGGATGATTGATTGCTCTCCTTTTGGCTGTCGAACAGGTTTTCACCTTATCATGTGGGGAAAACACAGTTCTACTGATATTGCCAAGGTGATTAAATCCAGCCTAGAAGAAATTGCAACTGGGATTACTTGGGAAGATGTTCCTGGAACAACTCTTGAATCCTGTGGGAACTATAAGGATCATAGCCTCTTTGCCGCCAAAGAATGGGCTCAATTGATTATTGATCAAGGGATTTCAGACGATCCTTTTAGTCGCCATGTCATCTGA

>Streptococcus pyogenes MGAS315

ATGACAAAAGAAGTTATCGTCGAAAGTTTTGAGCTAGATCATACTATTGTAAAGGCCCCTTATGTTCGTCTTATTTCTGAAGAATTTGGACCCAAGGGCGATCGTATTACAAATTTTGATGTTCGCCTAGTGCAGCCTAACCAAAATTCTATTGAAACAGCCGGTTTGCATACCATTGAACACTTACTTGCCAAGCTCATCCGCCAACGCATTGATGGGATGATTGATTGCTCTCCTTTTGGCTGTCGAACAGGTTTTCACCTTATCATGTGGGGAAAACACAGTTCTACTGATATTGCCAAGGTGATTAAATCCAGCCTAGAAGAAATTGCAACTGGGATTACTTGGGAAGATGTTCCTGGAACAACTCTTGAATCCTGTGGGAACTATAAGGATCATAGCCTCTTTGCCGCCAAAGAATGGGCTCAATTGATTATTGATCAAGGGATTTCAGACGATCCTTTTAGTCGCCATGTCATCTGA

>Streptococcus pyogenes strain: M3-b

ATGACAAAAGAAGTTATCGTCGAAAGTTTTGAGCTAGATCATACTATTGTAAAGGCCCCTTATGTTCGTCTTATTTCTGAAGAATTTGGACCCAAGGGCGATCGTATTACAAATTTTGATGTTCGCCTAGTGCAGCCTAACCAAAATTCTATTGAAACAGCCGGTTTGCATACCATTGAACACTTACTTGCCAAGCTCATCCGCCAACGCATTGATGGGATGATTGATTGCTCTCCTTTTGGCTGTCGAACAGGTTTTCACCTTATCATGTGGGGAAAACACAGTTCTACTGATATTGCCAAGGTGATTAAATCCAGCCTAGAAGAAATTGCAACTGGGATTACTTGGGAAGATGTTCCTGGAACAACTCTTGAATCCTGTGGGAACTATAAGGATCATAGCCTCTTTGCCGCCAAAGAATGGGCTCAATTGATTATTGATCAAGGGATTTCAGACGATCCTTTTAGTCGCCATGTCATCTGA

>Vibrio albensis strain NCIMB 41

ATGCCATTATTAGACAGTTTTACCGTCGATCATACCCGTATGAATGCACCGGCGGTGCGTGTTGCCAAAACCATGCAAACCCCAAAAGGGGATACGATTACCGTATTTGATTTGCGTTTTACTATGCCAAACAAAGATATCTTGTCTGAGCGCGGTATCCATACTCTAGAGCATCTCTACGCAGGCTTTATGCGTAATCACCTTAACGGCAGCCAAGTGGAGATCATTGATATTTCACCAATGGGTTGCCGTACAGGTTTCTACATGAGCTTGATTGGTGCGCCGACAGAACAGCAAGTGGCACAAGCATGGCTAGCCGCAATGCAAGATGTGCTGAAAGTTGAAAGCCAAGAGCAAATTCCTGAGCTGAATGAGTACCAGTGCGGCACTGCGGCGATGCACTCGCTCGAAGAAGCCAAAGCGATTGCGAAAAACGTGATTGCGGCAGGCATCTCGGTTAACCGTAACGATGAGTTGGCGCTGCCCGAATCTATGCTCAATGAGCTGAAGGTTCACTAA

>Vibrio cholerae strain E1320

ATGCCATTATTAGACAGTTTTACCGTCGATCATACTCGTATGAATGCACCGGCGGTGCGTGTTGCCAAAACCATGCAAACCCCAAAAGGGGATACGATTACCGTATTTGATTTGCGTTTTACTATGCCAAACAAAGATATCTTGTCTGAGCGCGGTATCCATACTCTAGAGCATCTCTACGCGGGCTTTATGCGCAATCACCTTAACGGCAGCCAAGTGGAGATCATCGATATTTCACCAATGGGTTGCCGTACAGGTTTCTACATGAGCTTGATTGGTGCGCCGACAGAACAGCAAGTGGCACAAGCATGGCTAGCCGCAATGCAAGATGTGTTGAAAGTTGAAAGCCAAGAGCAAATTCCTGAGCTGAATGAGTACCAGTGCGGCACTGCGGCGATGCACTCGCTCGAAGAAGCCAAAGCGATTGCGAAAAACGTGATTGCGGCAGGCATCTCGGTTAACCGTAACGATGAGTTGGCGCTGCCCGAATCTATGCTCAATGAGCTGAAGGTTCACTAA

>Vibrio parahaemolyticus AQ3810

ATGCCTTTACTCGATAGCTTCACCGTAGACCACACTCGCATGAATGCACCAGCAGTGCGTGTCGCGAAAACCATGCAAACTCCAAAAGGAGATACCATTACGGTTTTCGACTTACGCTTCACTGCGCCTAACAAAGACATTCTTTCAGAGAAAGGCATTCATACACTAGAGCACTTGTACGCAGGTTTCATGCGCAATCACCTGAATGGTGACAGTGTGGAAATCATTGATATCTCACCGATGGGATGTCGCACTGGTTTTTACATGAGTCTCATTGGTACGCCTTCTGAACAGCAAGTTGCAGACGCATGGCTTGCTTCTATGGAAGATGTGCTAAAAGTGGAAAGCCAGAACAAAATCCCTGAGCTAAATGAGTACCAGTGTGGTACTGCAGCGATGCATTCTTTAGAAGAAGCACAGCAAATTGCCAAGAACATTCTAGCCGCAGGTGTGTCTGTTAATAAAAACGACGAGTTGGCTTTGCCAGAATCAATGCTAAAAGAGCTGCGCGTAGACTAA

>Vibrio vulnificus strain CECT 4999

ATGCCATTATTAGATAGTTTTACCGTTGACCACACTCGCATGCATGCACCCGCGGTGCGTGTGGCATTTTCCATGCAGACGCCAAAAGGCGACACCATTACCGTGTTTGACTTACGTTTCACTGCACCTAACAAAGATATTTTGTCAGAGAAAGGCATTCACACGTTAGAGCACCTGTACGCAGGCTTTATGCGAAAGCATCTTAATGGCGCATCGGTTGAGATCATCGATATCTCACCGATGGGTTGTCGTACCGGTTTCTACATGAGCTTAATTGGTGCGCCGAGTGAGCAAGACGTGGCATCTGCGTGGACGGCTCCGATGGAAGATGTGTTGAAAGTGGAAAGCCAAAACAAGATCCCTGAGTTGAACGAGTATCAGTGTGGTACCGCGGCCATGCACTCGCTAGATGAAGCGAAGCAAATCGCGCAGAACATTCTGGCAGCACCAATTTCGGTGAATAAAAACGATGAACTGGCATTGCCAGAAGCGATGTTGAAAGAGCTCAAAGTGGATTAA

>Yersinia pestis biovar Microtus str. 91001

ATGCCATTATTGGATAGCTTTACCGTAGACCATACCATTATGAAAGCACCGGCAGTACGTGTCGCTAAGACGATGAAAACTCCTCATGGCGACGAGATAACGGTTTTTGATTTACGCTTCTGTGTGCCGAATAAAGAAGTGATGCCAGAGAAAGGGATCCATACGCTAGAGCACTTATTCGCTGGGTTTATGCGGGACCACCTCAATGGTGATGGTGTTGAAATTATTGATATCTCGCCGATGGGATGTCGCACGGGTTTCTACATGAGTCTGATAGGGACACCTGACGAGCAACGGGTTGCTGATGCCTGGAAAGCGGCAATGGCGGACGTGCTGAAGGTCACTGACCAGCGGAAGATCCCTGAGCTGAATGAATATCAGTGCGGGACTTATCATATGCACTCGCTGGAAGAAGCTCAGAGTATTGCTAAAGATATTCTTGACCGTGATGTGCGTATCAACCACAACGAAGAGTTGGCACTGCCGAAAGAGAAACTGACTGAATTGCATATTTAG

>Yersinia pestis CO92

ATGCCATTATTGGATAGCTTTACCGTAGACCATACCATTATGAAAGCACCGGCAGTACGTGTCGCTAAGACGATGAAAACTCCTCATGGCGACGAGATAACGGTTTTTGATTTACGCTTCTGTGTGCCGAATAAAGAAGTGATGCCAGAGAAAGGGATCCATACGCTAGAGCACTTATTCGCTGGGTTTATGCGGGACCACCTCAATGGTGATGGTGTTGAAATTATTGATATCTCGCCGATGGGATGTCGCACGGGTTTCTACATGAGTCTGATAGGGACACCTGACGAGCAACGGGTTGCTGATGCCTGGAAAGCGGCAATGGCGGACGTGCTGAAGGTCACTGACCAGCGGAAGATCCCTGAGCTGAATGAATATCAGTGCGGGACTTATCATATGCACTCGCTGGAAGAAGCTCAGAGTATTGCTAAAGATATTCTTGACCGTGATGTGCGTATCAACCACAACGAAGAGTTGGCACTGCCGAAAGAGAAACTGACTGAATTGCATATTTAG
